# Unusual light-driven amplification through unexpected regioselective photogeneration of five-membered azaheterocyclic AIEgen†

**DOI:** 10.1039/d0sc04725b

**Published:** 2020-10-19

**Authors:** Qiyao Li, Junyi Gong, Ying Li, Ruoyao Zhang, Haoran Wang, Jianquan Zhang, He Yan, Jacky W. Y. Lam, Herman H. Y. Sung, Ian D. Williams, Ryan T. K. Kwok, Min-Hui Li, Jianguo Wang, Ben Zhong Tang

**Affiliations:** Department of Chemistry, Hong Kong Branch of Chinese National Engineering Research Center for Tissue Restoration and Reconstruction, State Key Laboratory of Molecular Nanoscience, Division of Life Science, Department of Chemical and Biomedical Engineering and Institute for Advanced Study, The Hong Kong University of Science and Technology Clear Water Bay Kowloon Hong Kong China tangbenz@ust.hk; College of Chemistry and Chemical Engineering, Inner Mongolia Key Laboratory of Fine Organic Synthesis, Inner Mongolia University Hohhot 010021 China; Center for AIE Research, College of Materials Science and Engineering, Shenzhen University Shenzhen 518060 China; Chimie ParisTech, PSL University Paris, CNRS, Institut de Recherche de Chimie Paris Paris 75005 France; Center for Aggregation-induced Emission, SCUT–HKUST Joint Research Institute, State Key Laboratory of Luminescent Materials and Devices, South China University of Technology Guangzhou 510640 China

## Abstract

Developing versatile synthetic methodologies with merits of simplicity, efficiency, and environment friendliness for five-membered heterocycles is of incredible importance to pharmaceutical and material science, as well as a huge challenge to synthetic chemistry. Herein, an unexpected regioselective photoreaction to construct a fused five-membered azaheterocycle with an aggregation-induced emission (AIE) characteristic is developed under mild conditions. The formation of the five-membered ring is both thermodynamically and kinetically favored, as justified by theoretical calculation and experimental evidence. Markedly, a light-driven amplification strategy is proposed and applied in selective mitochondria-targeted cancer cell recognition and fluorescent photopattern fabrication with improved resolution. The work not only delivers the first report on efficiently generating a fused five-membered azaheterocyclic AIE luminogen under mild conditions *via* photoreaction, but also offers deep insight into the essence of the photosynthesis of fused five-membered azaheterocyclic compounds.

## Abstract 2

A multifunctional, AIE-based, fused five-membered azaheterocycle is photogenerated with light-driven amplification to combat the photobleaching issue and fabricate display materials.

## Graphical abstract

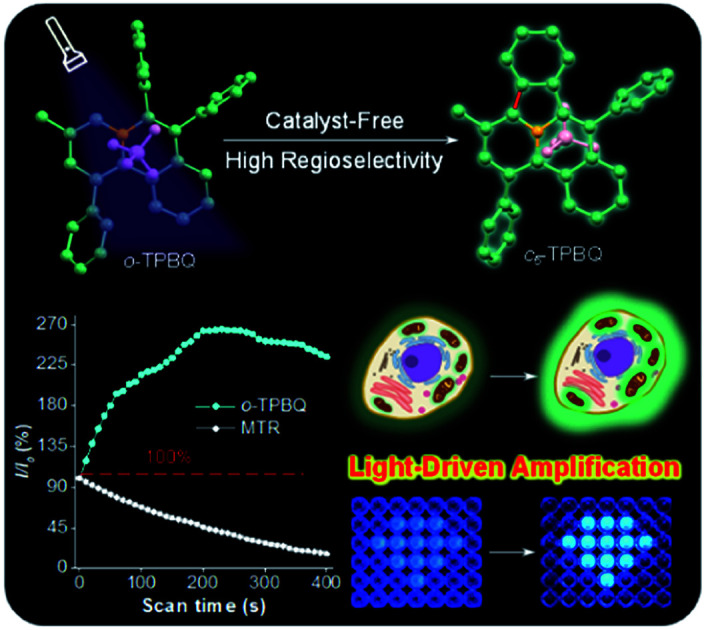

## Introduction

**Scheme 1:**
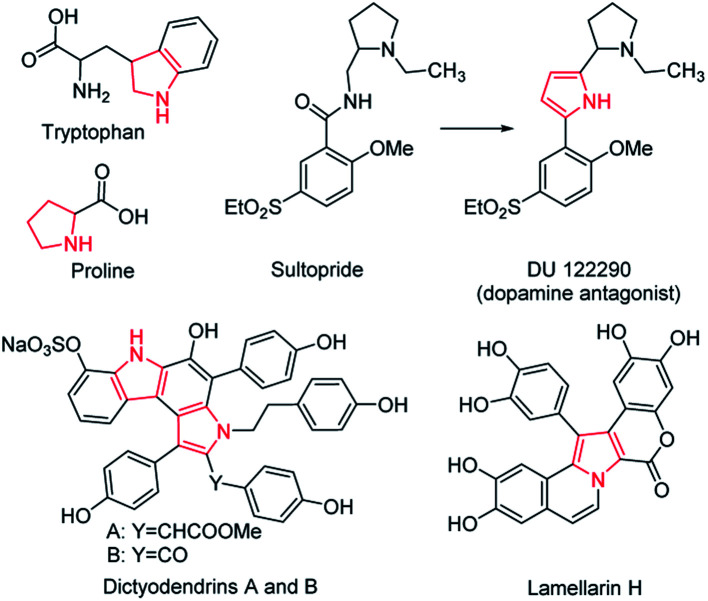
Representative examples of the bioactive five-membered azaheterocyclic compounds.

The five-membered ring compounds are widely distributed in nature, and are essential to life. In particular, those with N atoms possess significant structural features in many naturally occurring bioactive products, such as amino acids like tryptophan and proline (Scheme 1).^1,2^ Except for their vast distribution in natural products, five-membered azaheterocycles possess improved pharmacological activity.^3,4^ The superior antipsychotic activity and selectivity of DU 122290 compared to its lead compound (called sultopride) is a classic representative (Scheme 1).^5^ In particular, the fused five-membered azaheterocycles display remarkable biological activities.^6–8^ For example, dictyodendrins A and B isolated from the Japanese marine sponge, namely *Dictyodendrilla verongiformis*, show potent anti-telomerase activity, while Lamellarin H is an effective inhibitor towards both HeLa cells and HIV-1 integrase (Scheme 1).^9,10^ In addition, compounds containing five-membered azaheterocycles have found potential applications in various areas, including catalysis, agriculture, and electronics.^11–18^ Considering their great value in industrial applications and academic research, it is far from enough to obtain them by simple separation from natural products. Therefore, great effort has been put into exploring artificial synthetic methods for five-membered heterocycles, particularly the fused ones.^19–28^ During their development, one critical issue is that the metallic catalysts are often adopted for mediating an efficient synthesis, greatly raising the cost. Moreover, a time-consuming purification step needs to be performed to remove the remaining metal catalyst residue, as their trace presence may deteriorate the optoelectronic properties and cause cytotoxicity in the biological system.^29^ Another dilemma is that an oxygenic atmosphere is often required in the preparation to achieve better performance, inevitably resulting in byproducts at all kinds of levels, which result in poor selectivity and low efficiency. It is thus highly desirable to develop a versatile synthetic methodology with catalyst-free characteristics and high selectivity for the five-membered azaheterocyclic compounds.

From the information mentioned above, an ideal synthetic method should meet the following criteria: (1) as the multicomponent reaction may suffer from the tedious separation of various reactants and products, a single-component reaction without any additive is preferred. (2) The reaction can be carried out in aqueous medium under mild conditions with simple operation, which not only conforms to the postulation of green chemistry, but also is conducive to expanding applications of five-membered azaheterocycles. (3) The prepared products can be obtained with atom economy, in accordance with the sustainable development. In this regard, the photoreaction serves as a powerful protocol to construct various polycyclic aromatic hydrocarbons, attracting growing attention. Although impressive progress has been made, there are still two major challenges to overcome. Compared to the five-membered counterparts, products with six-membered rings are commonly reported due to the lesser strain required.^20^ In particular, cyclic compounds with a heteroatom are rarely prepared, and synthetic problems (including low yield and generation of various by-products) are often encountered. These challenges are mainly attributed to the fact that excited states are usually involved in the photoreaction. In addition, the intermediates in excited states are often not stable and easily react with oxygen or other species, giving undesired impurities.^30^ To solve the problems mentioned above and expand the application of five-membered azaheterocycles in living systems, much work has been done on the photogeneration of tetraphenylethylene (TPE) and its derivatives, which typically use aggregation-induced emission luminogens (AIEgens) with potential applications in bioimaging and theranostics.^31–36^ Although the resulting cyclic products were successfully employed in various fields including super-resolution imaging, organic optoelectronic devices and self-assemble materials, the N-containing fused five-membered heterocycle with AIE feature is rarely reported.^37–45^ Thus, generating AIE-based compounds with fused five-membered azaheterocycles *via* efficient and facile photoreaction is not only exceedingly challenging, but also exceptionally appealing.

In this contribution, we obtained an unexpected five-membered azaheterocyclic AIEgen, namely *c*_5_-TPBQ, with multiple functions *via* a clean, efficient and regioselective photoreaction. When *o*-TPBQ was merely irradiated by a hand-held UV lamp in the absence of any additive, it readily underwent photocyclization and afforded *c*_5_-TPBQ at high yield. Both compounds exhibited typical AIE behavior owing to the anion–π^+^ interactions. The photogeneration of *c*_5_-TPBQ could take place in aqueous media and common organic solvents. Specific mitochondrial imaging and selective cancer cell targeting were achieved at an ultralow nanomolar dye working concentration with unusual light-driven amplification, which was superior to commercial bioagents. Furthermore, making use of the light-dirven amplification, a 2D fluorescent pattern with enhanced contrast was fabricated in both solution and solid state under mild conditions.

## Results and discussion

As shown in Scheme S1,†*o*-TPBQ was readily achieved *via* a facile one-step synthetic route, according to the previously reported literature.^46,47^ Details of the experimental procedures are provided in the ESI.† The structure of *o*-TPBQ was well characterized, and confirmed by NMR and high-resolution mass spectroscopies (Fig. S1–S3†). Single crystals of *o*-TPBQ were successfully obtained in chloroform/MeOH mixtures by slow vapor diffusion (Table S1†).

**Fig. 1:**
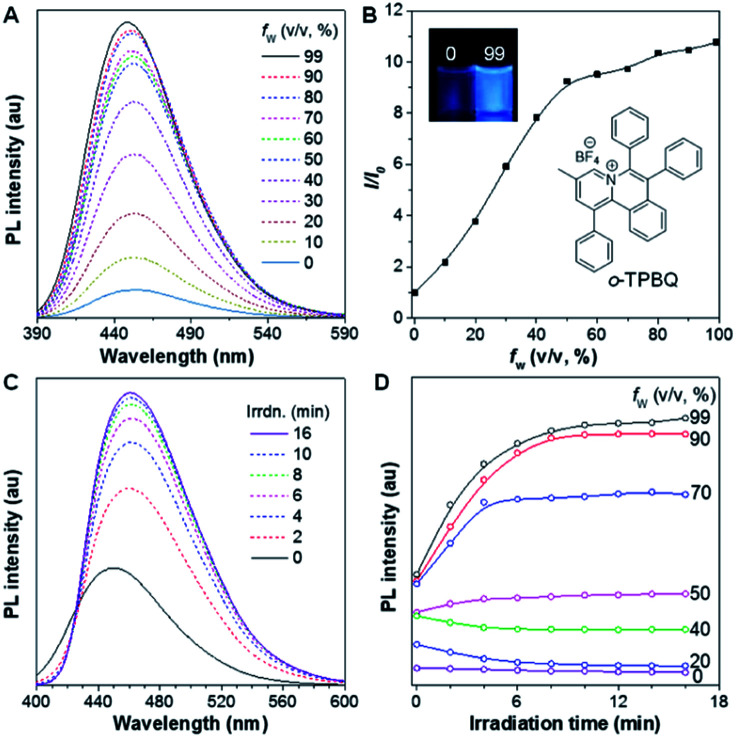
(A) PL spectra of *o*-TPBQ in DMSO/water mixtures with different water fractions (*f*_w_). (B) Plot of the relative emission intensity (*I*/*I*_0_) *versus f*_w_. Inset: fluorescence images of *o*-TPBQ in solution (*f*_w_ = 0%) and aggregate (*f*_w_ = 99%) states taken under 365 nm UV lamp. (C) PL spectra of *o*-TPBQ in DMSO/water mixtures with *f*_w_ = 99% at different irradiation times (irrdn.). (D) Plot of the emission peak of *o*-TPBQ in DMSO/water mixtures with different *f*_w_ and irradiation times. Excitation wavelength: 380 nm, concentration: 10 μM. Irradiation source: hand-held UV lamp at 365 nm.

**Fig. 2:**
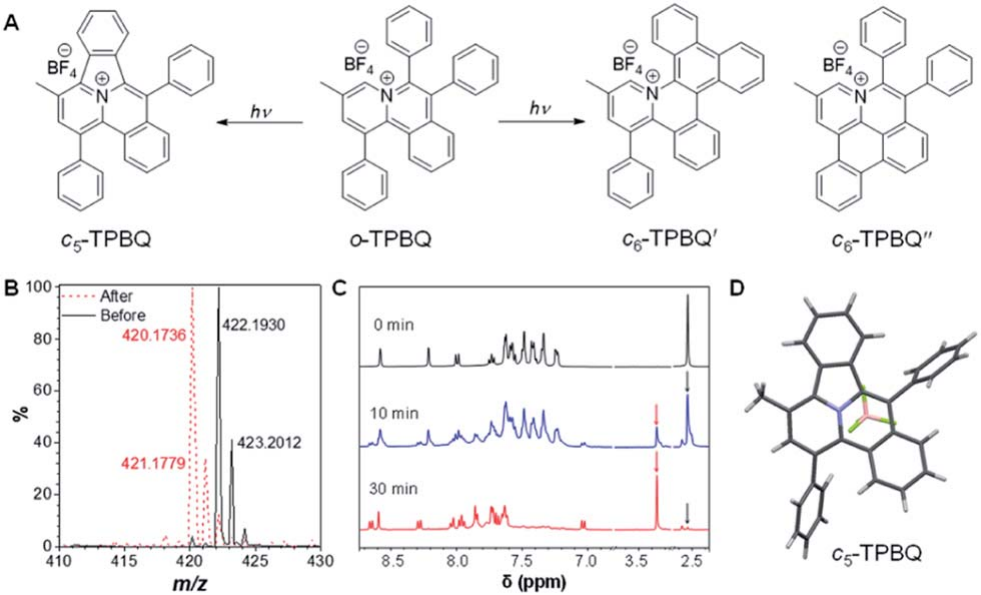
(A) Possible photogenerated products. (B) High-resolution mass spectra of *o*-TPBQ before and after UV irradiation from a hand-held UV lamp in DMSO solution. (C) ^1^H NMR spectrum of *o*-TPBQ under at different irradiation times. (D) Obtained single crystal structure of the photocyclized product (note: *c*_5_-TPBQ, instead of *c*_6_-TPBQ or *c*_6_-TPBQ′, was generated in the photoreaction).

After structural characterization, the emission behavior of *o*-TPBQ was investigated in DMSO/water mixtures by photoluminescence (PL) spectroscopy. As expected, *o*-TPBQ exhibited a typical AIE feature. As shown in Fig. 1A and B, *o*-TPBQ was weakly emissive in DMSO solution due to the consumption of the excited state energy by the active rotation of the phenyl rings. Upon gradually increasing the water fraction (*f*_w_), the emission intensity enhanced progressively owing to the restriction of molecular motion (RIM). Interestingly, a notable phenomenon was found wherein upon irradiating *o*-TPBQ in aggregate state (*f*_w_ = 99%) by a hand-held UV lamp, a brighter light blue fluorescence was observed by naked eye. As presented in Fig. 1C and D, with increased exposure time, the emission peak gradually redshifted from 450 nm to 460 nm, accompanied with significantly increased emission intensity. The emission became stronger with increasing irradiation time. To get a clearer map, we further measured the PL spectra of *o*-TPBQ at different *f*_w_ values under 365 nm UV irradiation (Fig. 1D). It was found that by prolonging the irradiation time, the emission intensity slightly weakened in mixtures with *f*_w_ below 50%. Afterward, the emission was enhanced gradually under UV irradiation. The emission was increasingly boosted with increasing *f*_w_. The absorption of *o*-TPBQ also varied with UV irradiation (Fig. S4†). Before irradiation, *o*-TPBQ exhibited a maximum absorption at 380 nm. Then, the absorption gradually shifted to a longer wavelength at 390 nm by prolonging the exposure time. Combining the results from the PL and UV-Vis measurements, we speculated that a product with greater conjugation was generated by the photoreaction. Taking the particular location of the N atom in *o*-TPBQ and the reported literature on the photoreaction into consideration, we proposed all of the possible products, as suggested in Fig. 2A. Basically, these compounds can be divided into two categories: the five-membered ring product (*c*_5_-TPBQ), although the chance is quite low; and the common six-membered ones, *c*_6_-TPBQ′ and *c*_6_-TPBQ′′.

To verify our hypothesis, HRMS and NMR spectra were utilized to determine the exact structure of the resultant product. After UV irradiation, the original peak at *m*/*z* 422.1930 (assigned to the mass of *o*-TPBQ minus the weight of tetrafluoroborate fragment) disappeared. Alternatively, a new peak at *m*/*z* 420.1736 emerged, indicating the loss of two hydrogen atoms (Fig. 2B). Subsequently, *in situ* dynamic NMR analysis was performed for better observation, as shown in Fig. 2C. Notably, by lengthening the exposure time, a new signal at *δ* 3.35 ppm (red arrow) appeared, while the signal at *δ* 2.50 ppm (black arrow) assigned to the methyl group of *o*-TPBQ gradually decreased. Meanwhile, a new double peak at *δ* 8.66 ppm emerged. A longer irradiation time (30 min) afforded the almost complete conversion of the unreacted signals. Although the results from the *in situ* HRMS and NMR analysis offered solid evidence of the occurrence of a photoreaction, we were still not able to tell the exact structure until the single crystal was achieved by slow vapor diffusion in chloroform/MeOH mixtures and analyzed crystallographically (Fig. 2D and Table S2†). Surprisingly, it turned out that only the five-membered cyclized product, *c*_5_-TPBQ, was obtained instead of the typical six-membered one (*c*_6_-TPBQ′ or *c*_6_-TPBQ′′). In addition, the structure of *c*_5_-TPBQ was confirmed by NMR and high-resolution mass spectroscopies (Fig. S5–S7†).

**Fig. 3:**
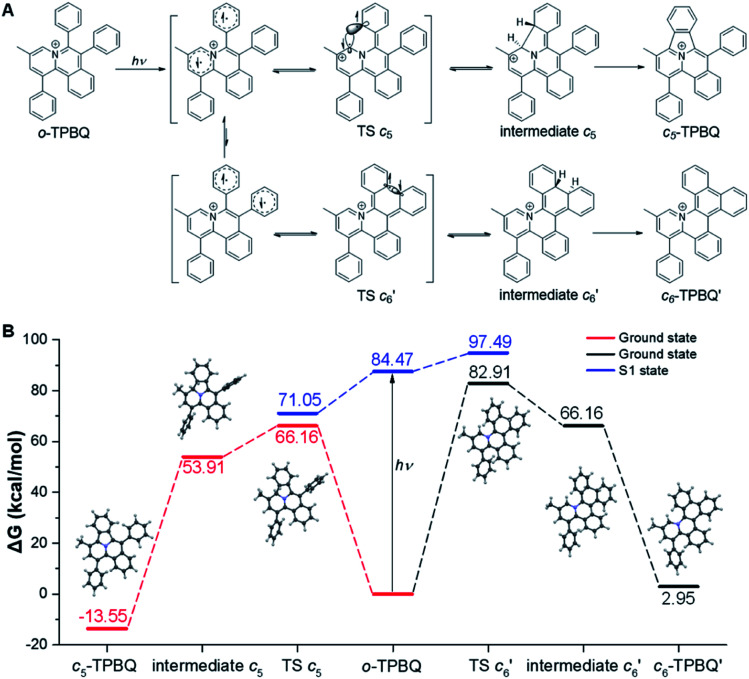
Proposed photoreaction mechanism of *o*-TPBQ. (A) Possible reaction pathway. (B) Gibbs free energy of the ground and S1 state of the five-membered ring product (*c*_5_-TPBQ) and six-membered ring product (*c*_6_-TPBQ′) formation process. The counteranions are omitted for clarity.

This unconventionally yet highly regioselective photocyclization greatly aroused our interest. Thus, we would like to have a deeper understanding of the mechanism. Here, a possible reaction pathway was proposed in forming the two kinds of products, as presented in Fig. 3A. After excitation, *o*-TPBQ generated a spin-polarized singlet excitation state. The exact spin-population distribution might lead the reaction to different products. TS *c*_5_ displayed one possibility: the spins mainly accumulated on the left benzene ring of the quinolinzinium structure and another neighboring free benzene ring, displaying a diradical behaviour. The TS *c*_5_ led to the intermediate *c*_5_ with a formed five-membered ring under dehydrogenation. The 
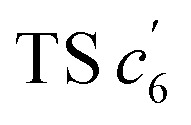
 revealed another possibility: the spins mainly accumulated on the two adjacent free benzene rings, which may yield intermediate 
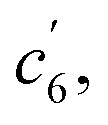
 which is one of the six-membered ring structures. The reaction pathway of *c*_6_-TPBQ′′ was analogous to that of *c*_6_-TPBQ′. To ascertain the likelihood of the two proposed scenarios in the photocyclization, density functional theory (DFT) calculations were performed based on both the ground and excited states. Fortunately, all encountered structures were confirmed, and their Gibbs free energies were calculated, respectively. As depicted in Fig. 3B and S8,† the free energy in the S1 excited state of *o*-TPBQ (84.47 kcal mol^−1^) is higher than that of TS *c*_5_ (71.05 kcal mol^−1^) and lower than that of 
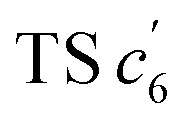
 (97.49 kcal mol^−1^), indicative of it favoring the five-membered ring pathway. Thus, the formation of the five-membered cyclic product is kinetically preferred. In addition, *c*_5_-TPBQ has a significantly lower free energy (−13.55 kcal mol^−1^) in the ground state than *c*_6_-TPBQ′ (2.95 kcal mol^−1^), suggesting that the highly selective formation of compound *c*_5_-TPBQ is thermodynamically controlled. Consequently, although the five-membered ring product appears impossible to form, in fact, it corresponds to the thermodynamics and kinetics. Except for the energy results obtained from the calculation, the preference for the five-membered ring formation can also be enlightened from the view of the molecular structure. For TS *c*_5_, one of the spins is stabilized by delocalization in a large conjugated core, thus reducing the total Gibbs free energy. Furthermore, calculations on the spin population analysis, which proved the radical character of the transition state, were consistent with the proposed reaction process (Fig. S9†). Formation of *c*_6_-TPBQ′′ is also unrealistic because this structure is unstable, and evolves back to the reactant *o*-TPBQ according to the DFT calculations. Thus, according to the calculation results and analysis above, *o*-TPBQ would go to TS *c*_5_ after excitation because of its lower free energy. It would then undoubtedly follow the five-membered ring path.

Subsequent experimental evidence gave further support for the reaction mechanism. The radical inhibitor 2,2,6,6-tetramethylpiperidine-1-oxyl (TEMPO) was added to the reaction system, and *in situ* NMR analysis was used to follow the reaction process. Here, the yield from *o*-TPBQ to *c*_5_-TPBQ was evaluated by the calculation of the conversion yield of the methyl peak of *o*-TPBQ at 2.50 ppm to that of *c*_5_-TPBQ at 3.35 ppm. As Fig. S10† shows, under 15 min UV exposure, compared to the control group with a yield of 45%, the TEMPO group had a much lower yield of 21%. When lengthening the irradiation time to 50 min, 47% yield of the TEMPO group was obtained compared to 85% of the Control group. From this observation, we can conclude that the reaction probably involves a radical process, in agreement with the proposed reaction mechanism. In addition, it is worth mentioning that many reported photoreactions required extra oxidant as the catalyst (such as iodine) for complete conversion, while our photocyclization is free of any additional catalyst or additive. Furthermore, we would like to explore whether the reaction would take place even without oxygen support (Fig. S11†). It was found that the conversion yield of the N_2_ group is negligibly lower than that of the control group, suggesting that the reaction could be carried out even under N_2_ atmosphere. Additionally, to evaluate the stability of the five-membered cyclized product, we intensified the irradiation conditions: a 500 W mercury lamp served as the irradiation source with added iodine as the oxidant, and the irradiation time was extended to 30 h (Fig. S12†). Much to our surprise, even under such highly intensive UV irradiation, the treated sample showed no spectral change compared to the pure *c*_5_-TPBQ. It turned out that no further cyclization occurred, and the product was not damaged at all. This suggested the extremely high regioselectivity of the photocyclization and the superb stability of the five-membered azaheterocyclic compound. Additionally, in order to prove that the synthesis of the five-membered azaheterocycles is a general strategy *via* photocyclization reaction, another two compounds, *o*-I and *o*-II, were prepared by removing the methyl group and changing the counterion, respectively (Fig. S13–S18, Tables S3 and S4†). After photoreaction under the same condition, the five-membered ring products, *c*_5_-I and *c*_5_-II were obtained, further indicating that the photoreaction may serve as the platform for five-membered azaheterocycle construction (Fig. S19–S22 and Table S5†).

**Fig. 4:**
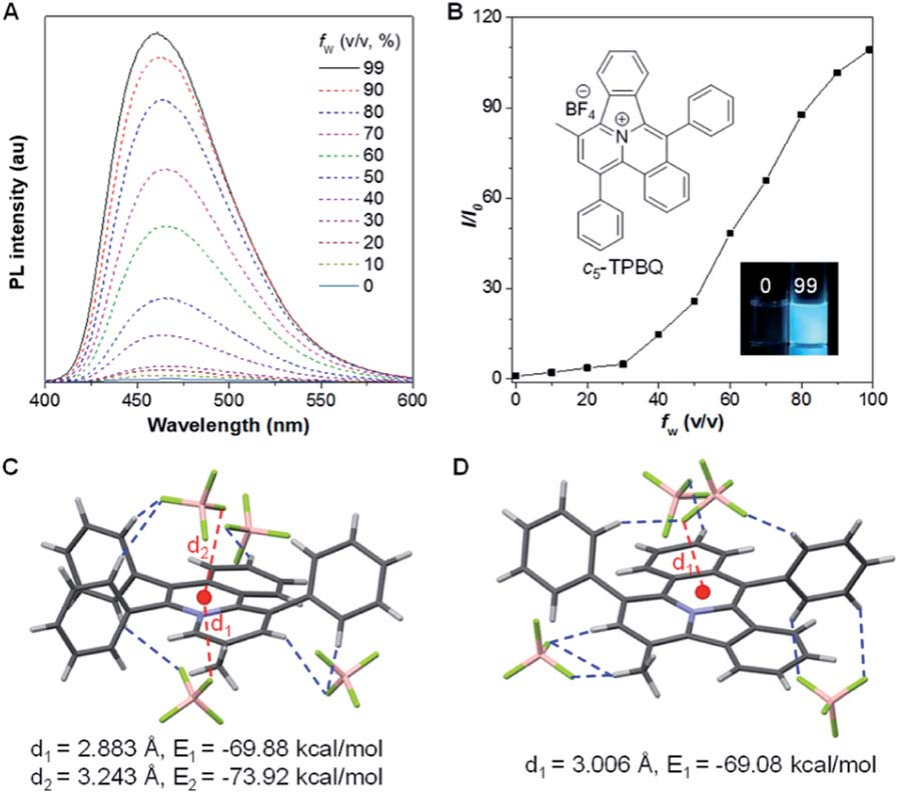
(A) PL spectra of *c*_5_-TPBQ in DMSO/water mixtures with different *f*_w_. (B) Plot of the relative emission intensity (*I*/*I*_0_) *versus f*_w_. Inset: fluorescence images of *c*_5_-TPBQ in solution (*f*_w_ = 0%) and aggregate (*f*_w_ = 99%) states under a hand-held 365 nm UV lamp. (C and D) Anion–π^+^ interactions (red) and the corresponding calculated energy, and hydrogen bond interactions (blue) in the single crystal structure of *o*-TPBQ (C) and *c*_5_-TPBQ (D).

After full elucidation of the mechanism of generating *c*_5_-TPBQ, we studied and compared the photophysical properties of the two compounds by UV-Vis and PL spectroscopies. The data are summarized in Table S6.† The absorption peaks of the two compounds in DMSO were found at 380 nm and 390 nm (Fig. S23†). The bathochromic shift of *c*_5_-TPBQ should be ascribed to its extended conjugation. For a deeper understanding, DFT calculations were performed based on the achieved single crystal structure. As suggested in Fig. S24,† the HOMO–LUMO energy gap of *c*_5_-TPBQ (3.92 eV) is smaller than that of *o*-TPBQ (4.30 eV), thus theoretically accounting for its red shift in absorption. The PL spectra of *c*_5_-TPBQ were measured in DMSO/water mixtures with different *f*_w_ (Fig. 4A). It is hardly emissive in DMSO solution with negligible fluorescence quantum yield (QY, *Φ*_soln_ = 1.1%), and then the enhanced PL signal was recorded with the gradual addition of water into the solution. The emission intensity in DMSO/water mixtures with *f*_w_ of 99% was boosted with 110-fold enhancement compared with that in the DMSO solution, demonstrating the AIE characteristic. Furthermore, it is noted that the QY of *c*_5_-TPBQ in *f*_w_ = 99% mixtures was measured to be 63.3%, which is almost three times higher than that of *o*-TPBQ (21.6%), thus leading to a higher *α*_A_IE value of *c*_5_-TPBQ (57.5) compared to that of *o*-TPBQ (12.0). In addition, for both compounds, the QY in the solid state was found to be decreased compared to that in the aggregate state. This may be ascribed to the relatively tight intermolecular packing that results from the self-assembly process and formed intramolecular noncovalent interactions in aqueous medium.^48^ Subsequently, the single crystal structures of *o*-TPBQ and *c*_5_-TPBQ were thoroughly analyzed. As presented in Fig. 4C, anion–π^+^ interactions between the fluorine atoms of tetrafluoroborate anions and the positively charged benzoquinoline core were found at the distance of 2.883 Å and 3.243 Å with calculated energies of −69.88 kcal mol^−1^ and −73.92 kcal mol^−1^, respectively. This effectively blocked the detrimental π–π stacking. Hydrogen bond interactions (blue dash) and intermolecular short contact interactions (Fig. S25†) were also observed in the crystal lattice, both of which restricted the rotation of the phenyl rings efficiently. For *c*_5_-TPBQ, analogous results were obtained. The anion–π^+^ interaction and the corresponding calculated energy were determined to be 3.006 Å and −69.08 kcal mol^−1^, respectively. The hydrogen bond and short contact interactions (Fig. S26†) also existed. Consequently, the anion–π^+^ interactions together with the multiple inter- and intra-molecular interactions (including hydrogen bonding and short contact interactions) account for the AIE characteristics of *o*-TPBQ and *c*_5_-TPBQ, which also agrees well with previous research studies.^46,49,50^ In addition, to gain insight into the increased QY in the aggregate state after cyclization, we checked and compared the crystal packing of the two compounds (Fig. S27†). Markedly, the arrangement of *c*_5_-TPBQ is relatively tight, forming a dimer-like packing, compared to the loosely packed *o*-TPBQ. SEM analysis was also performed (Fig. S28†). For *o*-TPBQ aggregates, a random and amorphous morphology was observed, whereas *c*_5_-TPBQ presented a regular-shaped and spherical morphology. Thus, the much stronger emission of *c*_5_-TPBQ in the aggregate state is possibly due to its tightly-packed arrangement and well-ordered morphology.

**Fig. 5:**
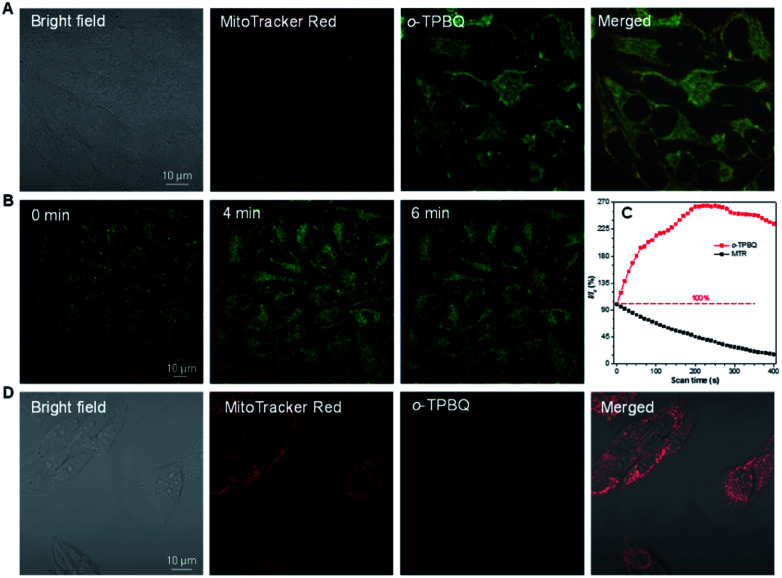
(A) Confocal images of HeLa cells constrained with MTR and *o*-TPBQ for 15 min. Concentration: 50 nM. (B) Confocal images of HeLa cells stained with *o*-TPBQ at different laser irradiation times. (C) Loss in fluorescence of HeLa cells stained with *o*-TPBQ and MTR with increasing scan times, respectively. (D) Confocal images of HLF normal cells stained with MTR and *o*-TPBQ for 15 min. Concentration: 1 μM.

Inspired by its positive charge and the previous research, we would like to explore the potential of *o*-TPBQ in bioimaging.^51,52^ First, the cytotoxicity of *o*-TPBQ and *c*_5_-TPBQ was evaluated by 3-(4,5-dimethyl-2-thiazolyl)-2,5-diphenyltetrazolium bromide (MTT) assay (Fig. S29†). For both *o*-TPBQ and *c*_5_-TPBQ groups, no significant drop of HeLa cell viability was observed with increased concentration after 24 h incubation. This suggested their acceptable biocompatibility, which provides the precondition in a biosystem. The cell imaging experiments were subsequently conducted *via* confocal laser scanning microscopy (CLSM). As presented in Fig. S30,† after incubating with HeLa cells at the concentration of 500 nM, the typical mitochondria network was clearly visualized with excellent contrast to the cell background. To further evaluate the specificity of *o*-TPBQ towards mitochondria, a colocalization experiment was performed by costaining with MitoTracker Red (MTR), which is a commercially available bioprobe for mitochondria. It turned out that the fluorescence signals from *o*-TPBQ and MTR merged well, with the Pearson correlation coefficient determined to be 0.89. To further verify the excellent cellular staining ability of *o*-TPBQ, we reduced the working concentration to as low as 50 nM. MTR was employed under the same condition for comparison. As shown in Fig. 5A, surprisingly, even at the ultralow concentration of 50 nM, the mitochondria were still clearly visualized by *o*-TPBQ, in sharp contrast to the faint and unclear fluorescence signal from MTR. Thus, our synthesized *o*-TPBQ was verified to be a mitochondria-targeted bioprobe with high specificity at an ultralow concentration, well surpassing the commercial fluorescence probe. To the best of our knowledge, the presented AIEgen holds the lowest working concentration compared with other previously reported mitochondria-targeting AIEgens working at micromolar level concentration.^53,54^ The photostability, on the other hand, considered of high essential for cell imaging and long-term tracking, was checked by continuous laser excitation and sequential scanning with confocal microscope. As illustrated in Fig. 5B and C, a moderately strong fluorescence from the *o*-TPBQ channel was observed before laser irradiation. Then, the emission intensity increased sharply upon laser excitation, reaching its peak at about 230 s. To more deeply understand the reason of the emission enhancement in the cell after laser irradiation, we successfully fished out the product from the cell medium. Compared to the samples under 500 W high-pressure mercury lamp irradiation, similar results were found in ^1^H NMR and HRMS analysis (Fig. S31 and S32†). With exposure to the laser, the signal at about *δ* 2.50 ppm assigned to the methyl group of *o*-TPBQ gradually transferred to a new signal at *δ* 3.35 ppm, and a new double peak at *δ* 8.66 ppm emerged (Fig. S31†). After laser irradiation, the peak at *m*/*z* 422.1909 is assigned to *o*-TPBQ, while the peak at *m*/*z* 420.1744 indicated the formed *c*_5_-TPBQ (Fig. S32†). Thus, evidence from NMR and HRMS under biological conditions both verified that *c*_5_-TPBQ was generated under laser irradiation during cell imaging. In addition, the photostability test on *c*_5_-TPBQ suggested its satisfactory photostability (Fig. S33†). Thus, this indicated that the unique light-driven amplification ascribes the formed *c*_5_-TPBQ *via* photocyclization reaction and its excellent photostability. In contrast, the emission of MTR was significantly quenched after 230 s irradiation under the same circumstances. This *in situ* light-driven amplification strategy, owing to the accessible photoreaction and the exceptional fluorescence feature of the cyclized product, can provide a potential application in cell tracking and solving the photobleaching problem. Additionally, inspired by the previous report that AIEgens (with both inherent positive charge and mitochondrial-targeting capability) could be promising candidates for differentiating cancer cells from normal cells, cell imaging experiments using HepG2 cancer cells and normal cells (including HLF and COS-7 cells) were carried out under the same experimental conditions as those of HeLa cells.^55^ Interestingly, for both COS-7 and HLF normal cells, no fluorescence from *o*-TPBQ was observed at the concentration of 50 nM (Fig. S34†). Even when increasing the concentration to 1 μM, barely any signal was recorded for both normal cell lines (Fig. 5D and S35†). For HepG2 cancer cells (Fig. S36 and S37†), the obtained results were similar to those of HeLa cells: *o*-TPBQ can target mitochondria with high specificity in cancer cells at the low concentration. Therefore, all of these results verified that the easily prepared AIEgen, *o*-TPBQ, working at nanomolar level concentration, was able to serve as an extraordinary bioimaging agent in specific mitochondria-targeting and selective cancer cell accumulation with the exceptional light-driven amplification achieved in photogeneration.

**Fig. 6:**
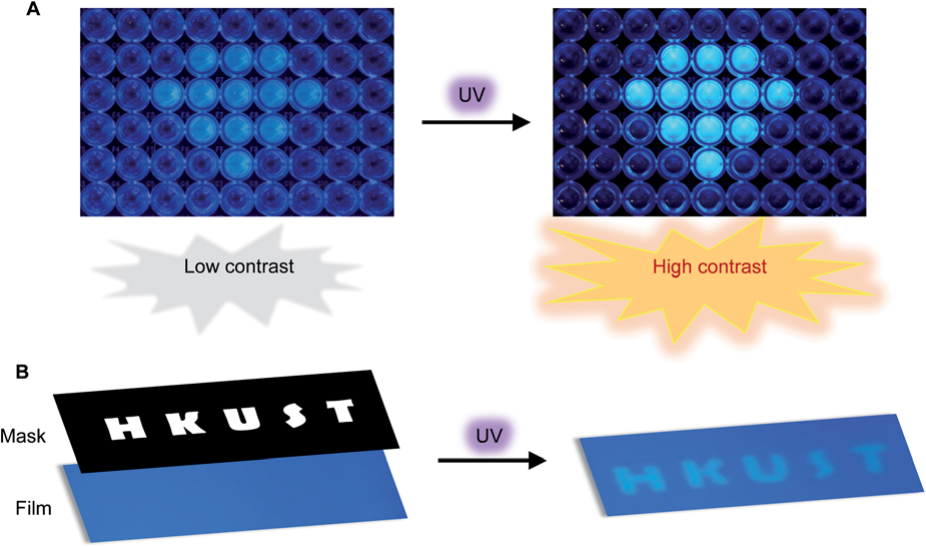
(A) Photopattern in DMSO/H_2_O mixtures with *f*_w_ = 99% (the “diamond”) and *f*_w_ = 0% (background). Concentration: 10 μM. (B) Photopattern materials for printing “HKUST”. Irradiation source: a 365 nm hand-held UV lamp.

Encouraged by the unusual light-driven amplification observed and the easily accessible photocyclization of *o*-TPBQ, display materials with improved signal-to-background ratio (S/B) were fabricated on solution and solid support, respectively. As presented in Fig. 6A, 99% aqueous solvent of *o*-TPBQ was filled in the “diamond” pattern, while the background holes were filled with *o*-TPBQ in pure DMSO solution. Without UV exposure, the “diamond” could merely be observed in a low contrast. Upon irradiation from a hand-held UV lamp for a few seconds, however, the “diamond” “shone” immediately. As shown in Fig. S38A,† the most noteworthy feature of this “turn-up” process is the improvement of the signal-to-background ratio (from 2.61 to 8.14) realized by simultaneously enhancing the fluorescence signal and reducing interference from the background. Subsequently, a writable photopatterning material was built on a solid support. As shown in Fig. 6B and S38B,† a TLC plate coated with *o*-TPBQ served as the matrices. Following several seconds irradiation from a hand-held UV lamp under the covered photomask, the fluorescence “HKUST” pattern was successfully generated with S/B = 1.35.

Impressed by the broad applications of *c*_5_-TPBQ obtained by the facial and efficient photosynthesis, we proposed a rational design strategy for the five-membered azaheterocycles: the reactive site of photocyclization tends to locate at the electron-poor region of a molecule, such as the position *ortho* or *para* to the N atom of pyridinium. Thus, if the spatial structure permits, introducing a nitrogen cation into an aromatic system can increase the possibility of five-membered ring formation. In addition, it is delightful to find that the reactive-site principle is supported by previous work.^37^ In this way, under the guidance of the reactive-site principle, photoreaction may serve as a platform for constructing five-membered azaheterocyclic compounds.

## Conclusion

In summary, an unexpected multifunctional five-membered azaheterocycle, *c*_5_-TPBQ, was photogenerated with unconventional but highly specific regioselectivity. The yield can reach up to unity without the participation of an oxidant or catalyst, conforming to the atom economy principle. Except for organic solvents, the photoreaction can proceed equally in aqueous medium, meeting the requirements of green chemistry. A possible reaction mechanism for the formation of the five-membered ring product instead of six-membered counterparts was proposed, which was clearly rationalized by theoretical and experimental studies. The physical and optical properties of the *o*-TPBQ and *c*_5_-TPBQ were fully investigated by photoluminescence, UV-Vis absorption, single crystal X-ray, theoretical calculation and SEM analysis. Notably, the resultant *c*_5_-TPBQ displayed an enhanced AIE feature compared to its reactant *o*-TPBQ. Working as an attractive bioimaging agent, *o*-TPBQ exhibited several exceptional merits, including satisfied biocompatibility, specific mitochondria-targeting, nanomolar level working concentration, and selective cancer cell differentiation. Notably, a light-driven amplification phenomenon was observed as a result of photoreaction upon laser excitation in bioimaging, suggestive of its strong resistance to photobleaching. Taking advantage of the simple and mild reaction conditions, as well as the unique light-driven amplification, fluorescent 2D photopatterns were fabricated in the solution and solid state, respectively. Finally, we creatively put forward a simple reactive-site principle for photogenerating five-membered azaheterocycles. Therefore, this work paves the way toward efficiently constructing fused five-membered azaheterocyclic compounds with unique fluorescent properties under simple and mild conditions, which can find an array of applications in biological and optoelectronic fields.

## Experimental section

### Materials and instrumentation

All chemicals were purchased from J&K Chemistry, Sigma-Aldrich and TCI, and used directly without further purification. Cells were obtained from the American Type Culture Collection.

^1^H NMR and ^13^C NMR spectra were recorded with a Bruker ARX 400 NMR spectrometer. High-resolution mass spectra (HRMS) were recorded on a GCT premier CAB048 mass spectrometer operating in a MALDI-TOF mode. UV-Vis absorption spectra were recorded on a PerkinElmer Lambda 365 Spectrophotometer. Photoluminescence (PL) spectra were recorded on a Fluorolog®-3 Spectrofluorometer. The absolute fluorescence quantum yield was measured using a Hamamatsu quantum yield spectrometer C11347 Quantaurus QY. The lifetime was measured on an Edinburgh FLS980 fluorescence spectrophotometer equipped with a xenon arc lamp (Xe900). Single crystal X-ray diffraction was performed on a *D*/max-2550 PC X-ray diffractometer (XRD; Rigaku, Cu-Kα radiation). The crystal data were collected on an Oxford Diffraction Xcalibur Atlas Gemini ultra instrument. The scanning electron microscope image was taken using a JSM-6390 scanning electron microscope. The fluorescence images were taken by confocal laser scanning microscope (CLSM) (Zeiss, Germany).

### Synthesis of *c*_5_-TPBQ

To a round-bottom flask was added *o*-TPBQ (30 mg) dissolved in CH_3_CN solution. The resulting solution was stirred under irradiation from a 500 W high-pressure mercury vapor lamp for 1 h for complete reaction. The crude product was purified by silica gel column chromatography with DCM : MeOH (5 : 1, v/v) in 94% yield. ^1^H NMR (400 MHz, CD_2_Cl_2_), *δ* (ppm): 8.66 (d, *J* = 8.0 Hz, 1H), 8.60 (s, 1H), 8.29 (d, *J* = 8.7 Hz, 1H), 8.07–7.92 (m, 3H), 7.85 (d, *J* = 6.5 Hz, 3H), 7.77–7.68 (m, 5H), 7.66–7.60 (m, 4H), 7.04 (d, *J* = 8.2 Hz, 1H), 3.37 (s, 3H). ^13^C NMR (100 MHz, CD_2_Cl_2_), *δ* (ppm): 145.01, 139.68, 138.36, 138.24, 137.16, 135.73, 133.44, 132.84, 132.57, 132.30, 132.27, 131.79, 131.36, 130.82, 130.60, 130.29, 130.17, 130.05, 129.99, 129.32, 128.91, 128.84, 128.29, 125.13, 124.84, 124.56, 19.71. HRMS (MALDI-TOF): *m*/*z*: [M − BF_4_]^+^ calcd for C_32_H_22_N^+^: 420.1747; found: 420.1774.

### Theoretical calculation

All calculations were done on the quantum mechanics package ORCA 4.1. (1) The initial structures were generated from the crystal diffraction data with optimized proton coordination under BLYP/def2-SVP level with density fitting approximation. The transition states (TS) were found with QST (Berny) algorithm with initial guesses generated by Prof. Grimme's XTB software. (2) The single point energies of all encountered geometries were calculated by adding the transition energy from the TD-DFT calculation (PBE0/def2-SVP) to the ground state Gibbs free energy (PBE0/def2-SVP). The spin population analysis was performed on the multifunctional wavefunction analysis software Multiwfn 3.6. (3).

## Author contributions

B. Z. T., J. W. and Q. L. conceived the original idea for this study. Q. L. synthesized and characterized the molecules. Q. L. did the photoreaction. J. Z. and H. Y. provided the photoreactor. J. G. performed theoretical calculations. Y. L. and R. Z. did the cell imaging experiments. H. W. did the photopattern. H. H. Y. S. and I. D. W. did the crystal analysis. B. Z. T. supervised the whole process. B. Z. T., J. W., Q. L., J. G., R. T. K. K and M. H. L. analyzed the data and participated in the discussion. Q. L., J. W. and J. W. Y. L. revised the manuscript. Q. L. and J. W. wrote the manuscript with comments from all authors.

## Conflicts of interest

There are no conflicts to declare.

## Supplementary Material

SC-012-D0SC04725B-s001

SC-012-D0SC04725B-s002
