# Supplementary material for: Unusual light-driven amplification through unexpected regioselective photogeneration of five-membered azaheterocyclic AIEgen
Source: Chem Sci. 2020 Oct 19;12(2):709–17. doi: 10.1039/d0sc04725b (PMC8179000; doi:10.1039/d0sc04725b)
Supplement: SC-012-D0SC04725B-s001 [file SC-012-D0SC04725B-s001.pdf]

Electronic Supplementary Material (ESI) for Chemical Science.
This journal is © The Royal Society of Chemistry 2020

Supporting Information

Unusual Light-Driven Amplification through Unexpected Regioselective Photogeneration of Five-Membered Azaheterocyclic AIEgen

Qiyao Li, [†a] Junyi Gong, [†a] Ying Li, [ac] Ruoyao Zhang, [a] Haoran Wang, [a] Jianquan Zhang, [a] He Yan, [a] Jacky W. Y. Lam, [a] Herman H. Y. Sung, [a] Ian D. Williams, [a] Ryan T. K. Kwok, [a] Min-Hui Li, [d] Jianguo Wang* [b] and Ben Zhong Tang* [ace]

Synthesis of *o*-TPBQ:

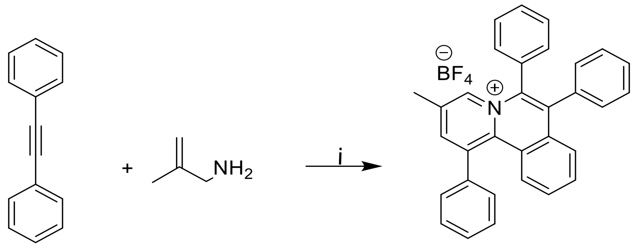

Scheme S1. Synthetic route to *o*-TPBQ: (i) $NaBF_4$, $[Cp^*RhCl_2]_2$, $Cu(OAc)_2{\cdot}H_2O$, MeOH, 130 °C, 6 h.

To a 25 mL pressure vial were added methallyl amine (0.4 mmol), diphenylacetylene (1.2 mmol), copper acetate (2 mmol), $[Cp^*RhCl_2]_2$ (0.02 mmol), tetrafluoroboric acid (48% in water, 0.6 mmol) and methanol. The resulting solution was stirred at 130 °C overnight, dried over anhydrous $MgSO_4$. The crude product was purified by silica gel column chromatography with DCM:MeOH (10:1, *v*:*v*) in 85% yield. ^{1}H NMR (400 MHz, CD_2Cl_2), δ (ppm): 8.58 (s, 1H), 8.21 (d, *J* = 1.8 Hz, 1H), 8.00 (d, *J* = 8.7 Hz, 1H), 7.73 (t, *J* = 7.7 Hz, 1H), 7.68 – 7.53 (m, 14H), 7.54 – 7.32 (m, 9H), 7.26 – 7.22 (m, 2H), 2.53 (s, 3H). ^{13}C NMR (100 MHz, CD_2Cl_2), δ (ppm): 144.56 , 141.71 , 140.27 , 139.75 , 138.02 , 137.36 , 134.54 , 134.33 , 134.19 , 133.49 , 133.07 , 131.30 , 131.10 , 130.41 , 130.18 , 130.14 , 129.86 , 129.69 , 129.66 , 128.76 , 128.64 , 128.42 , 128.38 , 127.30 , 124.93 , 18.58. HRMS (MALDI-TOF): *m/z:* $[M-BF_4]^+$ calcd for $C_{32}H_{24}N^+$: 422.1903; found: 422.1948.

Synthesis of *o*-I and *o*-II:

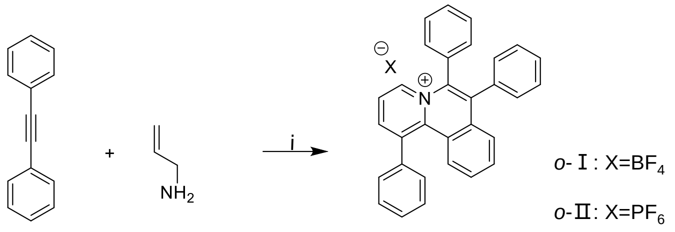

Scheme S2. Synthetic route to *o*-I and *o*-II: (i) NaX, $[Cp^*RhCl_2]_2$, $Cu(OAc)_2{\cdot}H_2O$, MeOH, 130 ℃, 6 h.

To a 25 mL pressure vial were added allyl amine (0.4 mmol), diphenylacetylene (1.2 mmol), copper acetate (2 mmol), $[Cp^*RhCl_2]_2$ (0.02 mmol), NaX (0.6 mmol) and methanol. The resulting solution was stirred at 130 °C overnight, dried over anhydrous $MgSO_4$. The crude product was purified by silica gel column chromatography with DCM:MeOH (10:1, *v*:*v*).

For compound *o*-Ⅰ : ^{1}H NMR (400 MHz, DMSO-d_6), δ (ppm): δ 8.86 (dd, J = 6.8, 1.3 Hz, 1H), 8.60 (dd, J = 7.6, 1.3 Hz, 1H), 8.14 (t, J = 7.2 Hz, 1H), 7.95 (d, J = 8.7 Hz, 1H), 7.87 (ddd, J = 8.2, 7.0, 1.1 Hz, 1H), 7.67 (s, 5H), 7.58 – 7.25 (m, 12H). ^{13}C NMR (100 MHz, DMSO-d_6), δ (ppm): 142.79, 140.02, 139.39, 138.45, 136.71, 135.46, 134.36, 133.43, 131.62, 131.00, 130.14, 130.05, 129.91, 129.51, 129.45, 129.40, 128.69, 128.36, 128.29, 128.20, 126.56, 124.60, 123.41. HRMS (MALDI-TOF): *m/z:* $[M-BF_4]^+$ calcd for $C_{31}H_{22}N^+$: 408.1447; found: 408.1760.

For compound *o*-Ⅱ: ^{1}H NMR (400 MHz, CD_2Cl_2), δ (ppm): 8.88 (d, J = 6.9 Hz, 1H), 8.40 (d, J = 7.5 Hz, 1H), 8.10 (d, J = 8.7 Hz, 1H), 7.95 (t, J = 7.2 Hz, 1H), 7.81 (t, J = 7.7 Hz, 1H), 7.66 (q, J = 7.6, 5.8 Hz, 6H), 7.58 – 7.45 (m, 6H), 7.41 – 7.29 (m, 5H). ^{13}C NMR (100 MHz, CD_2Cl_2), δ (ppm): 142.98, 141.43, 139.97, 138.65, 136.22, 134.36, 134.16, 133.99, 131.37, 131.28, 130.87, 130.61, 130.38, 130.30, 130.18, 129.12, 128.99, 128.86, 128.82, 127.75, 125.21, 123.25. HRMS (MALDI-TOF): *m/z:* $[M-PF_6]^+$ calcd for $C_{31}H_{22}N^+$: 408.1447; found: 408.1772.

Photosynthesis of *c*5-I and *c*5-II:

To a round-bottom flask was added c_5-I or c_5-II (30 mg) dissolved in CH_3CN solution. The resulting solution was stirred under irradiation from a 500 W high-pressure mercury vapor lamp for 1 h for complete reaction. The crude product was purified by silica gel column chromatography with DCM:MeOH (5:1, *v*:*v*).

***c*5-Ⅰ**: ^1H NMR (400 MHz, Acetonitrile-d_3), δ (ppm): 9.17 (d, J = 8.1 Hz, 1H), 8.82 (d, J = 8.1 Hz, 1H), 8.72 (d, J = 7.9 Hz, 1H), 8.28 (d, J = 8.7 Hz, 1H), 8.07 – 8.00 (m, 2H), 7.97 – 7.88 (m, 4H), 7.78 – 7.64 (m, 9H), 6.98 (d, J = 8.1 Hz, 1H). HRMS (MALDI-TOF): *m/z:* $[M-BF_4]^+$ calcd for $C_{32}H_{20}N^+$: 406.1590; found: 406.1602

***c*5-Ⅱ**: ^1H NMR (400 MHz, Acetonitrile-d_3), δ (ppm): 9.13 (d, J = 8.0 Hz, 1H), 8.79 (d, J = 8.1 Hz, 1H), 8.68 (d, J = 7.9 Hz, 1H), 8.25 (d, J = 8.7 Hz, 1H), 8.05 – 7.97 (m, 2H), 7.89 (dd, J = 19.3, 7.4 Hz, 4H), 7.76 – 7.63 (m, 9H), 6.95 (d, J = 8.1 Hz, 1H). HRMS (MALDI-TOF): *m/z:* $[M-PF_6]^+$ calcd for $C_{32}H_{20}N^+$: 406.1590; found: 406.1604

***In situ* NMR measurement**

For better observation and accurate analysis, a 500 W high-pressure mercury lamp was used as the irradiation source for *in situ* ^1H NMR measurement (Fig. 2C, S10 and S11).

In situ NMR measurement under biological conditions, working concentration of *o*-TPBQ was amplified for clear analysis. The laser source was the same as that in imaging experiments.

Sample preparation

A stock solution of *o*-TPBQ in DMSO with a concentration of 10 mM was prepared and stored in the 4 °C fridge.

Cell culturing and staining

HeLa, HepG2, COS-7 and HLF cells were cultured in dulbecco's modified eagle medium (DMEM), respectively. All the cells were grown in the media which supplied with 10% fetal

bovine serum (FBS), 100 U/mL penicillin and 100 μg/mL streptomycin in a humidified incubator at 37 °C with 5% CO_2 and subcultured every two or three days.

The cells were seeded and grown overnight on a 35 mm petri dish with a cover slip. The cells were incubated with *o*-TPBQ at the required concerntration (stock solution diluted to a 1 mL culture medium) for 15 min. To investigate the distribution of *o*-TPBQ, Mito Tracker Red (MTR) was used to stain the mitochondria under the same condition. The cells were imaged under a confocal microscope (Zeiss LSM 800 laser scanning confocal microscope) using proper excitation and emission filters for each dye: for *o*-TPBQ, the excitation filter was 405 nm and the emission filter 410−600 nm; for MTR, the excitation filter was 561 nm and the emission filter 565−700 nm. For photostability experiments, cells incubated with different dyes were continuously irradiated with confocal lasers (for *o*-TPBQ and c_5-TPBQ, laser wavelength: 405 nm; for MTR, laser wavelength: 561 nm; laser power: 1.2 %). Continuous scans (10 s per scan) were taken.

Cell viability *via* MTT Assay

The cells were grown on a 96-well plate at a density of 10000 cells per well and incubated for 24 h. After being incubated with *o*-TPBQ and c_5-TPBQ and at different concentrations, respectively. The cell incubated 24 h and then changed the new DMEM medium. 100 µL of fresh DMEM medium containing 10 µL 3-(4,5-Dimethylthiazol-2-yl)-2,5-Diphenyltetrazolium Bromide) (MTT) (5 mg/mL) solution was added to each well after removal of the cell medium, and the cells were incubated for 4 h. 100 μL DMSO was added to each well after remove the MTT solution. The absorbance at 570 nm was recorded by a microplate reader (Perkin-Elmer Victor3t).

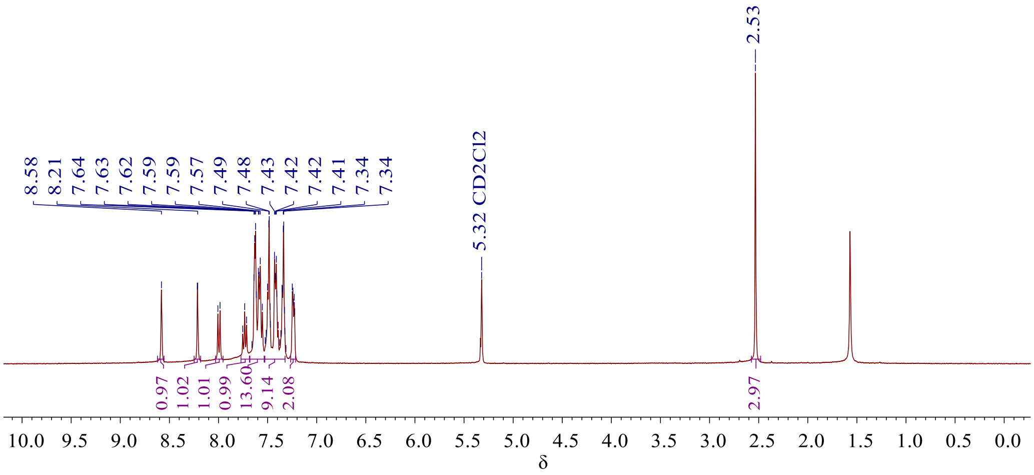

Figure S1. ^{1}H NMR spectrum of compound *o*-TPBQ in CD_2Cl_2.

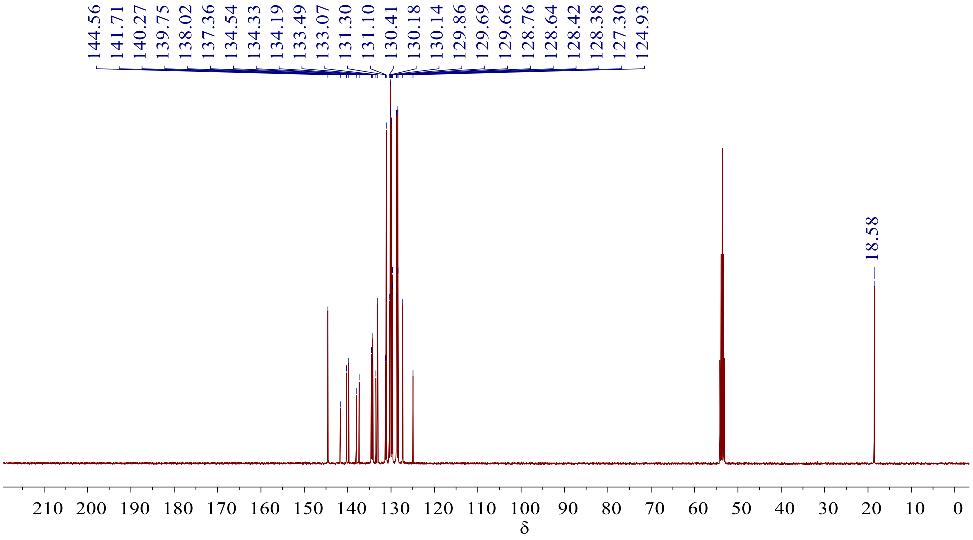

Figure S2. ^{13}C NMR spectrum of compound *o*-TPBQ in CD_2Cl_2.

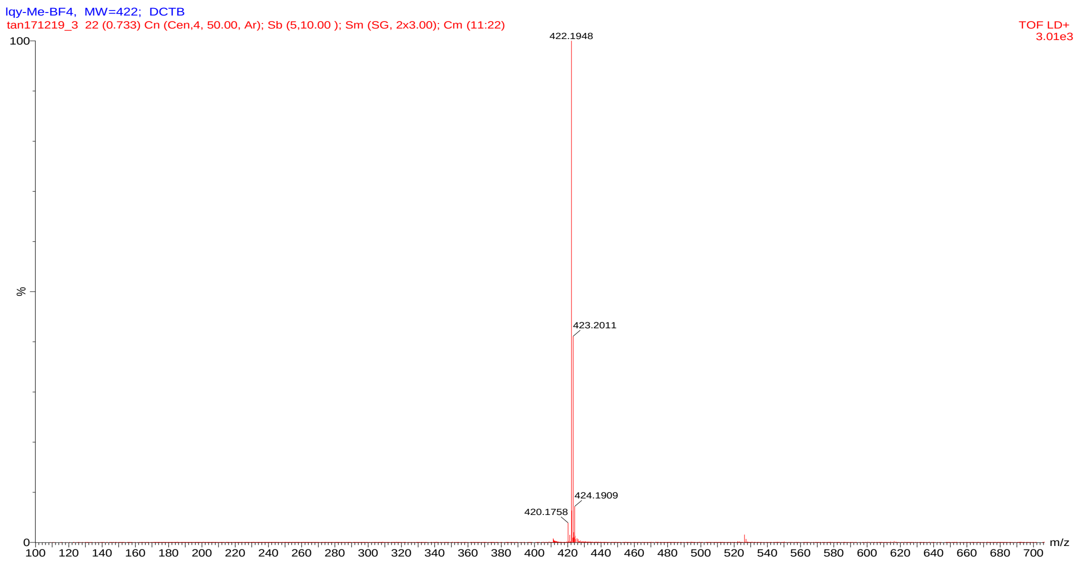

Figure S3. HRMS spectrum of compound *o*-TPBQ.

Table S1. Crystallographic and structural refinement data of *o*-TPBQ.[a]

Empirical formula	$C_{32}H_{24}BF_4N$
Formula weight	509.33
Temperature (K)	199.99(10)
Wavelength (Å)	1.54184
Crystal system	Monoclinic
space group	P 1 21/c 1
a (Å)	12.5860(2)
b (Å)	15.1416(2)
c (Å)	13.4977(2)
α (°)	90
β (°)	97.2530(10)
γ (°)	90
Volume (Å^3)	2551.70(7)
Z	4
θ range (°)	3.540 to 64.999
Index ranges	14<=h<=8, 17<=k<=13, 15<=l<=15

[a]Crystallographic data for the structures reported in this paper have been deposited with the Cambridge Crystallographic Data Centre as supplementary publication no. CCDC: 2008533 for *o*-TPBQ.

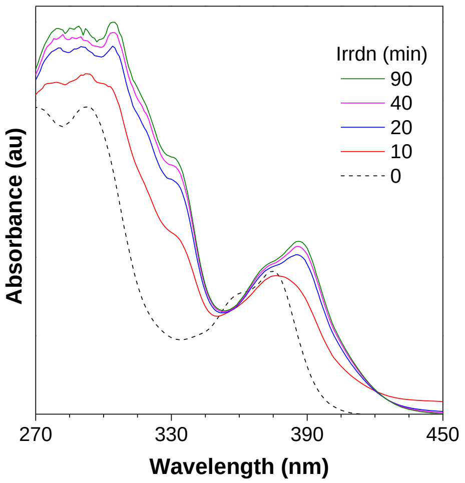

Figure S4. Time-dependent UV−vis spectra of *o*-TPBQ in DMSO/H_2O mixtures with f_W = 99% upon 365 nm UV irradiation from a hand-held UV lamp. Concentration: 20 μM.

Table S2. Crystallographic and structural refinement data of c_5-TPBQ.[a]

Empirical formula	$C_{32}H_{22}BF_4N$
Formula weight	507.31
Temperature (K)	100.01(10)
Wavelength (Å)	1.54184
Crystal system	Orthorhombic
space group	Pbca
a (Å)	15.62776(16)
b (Å)	16.52398(16)
c (Å)	18.67576(19)
α (°)	90
β (°)	90
γ (°)	90
Volume ($Å^3$)	4822.69(8)
Z	8
θ range (°)	4.558 to 67.496
Index ranges	-18<=h<=18, -19<=k<=19, -22<=l<=15

[a]Crystallographic data for the structures reported in this paper have been deposited with the Cambridge Crystallographic Data Centre as supplementary publication no. CCDC: 2008534 for c_5-TPBQ.

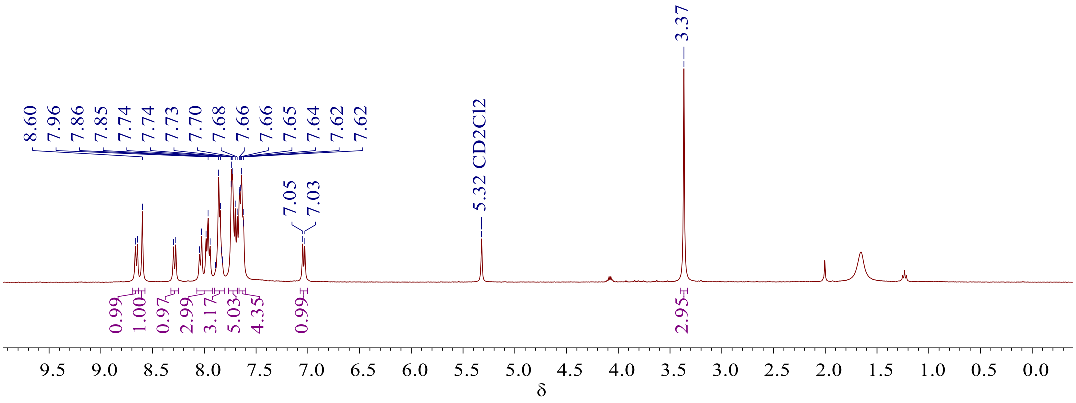

Figure S5. ^{1}H NMR spectrum of compound *c*$_5$-TPBQ in CD_2Cl_2.

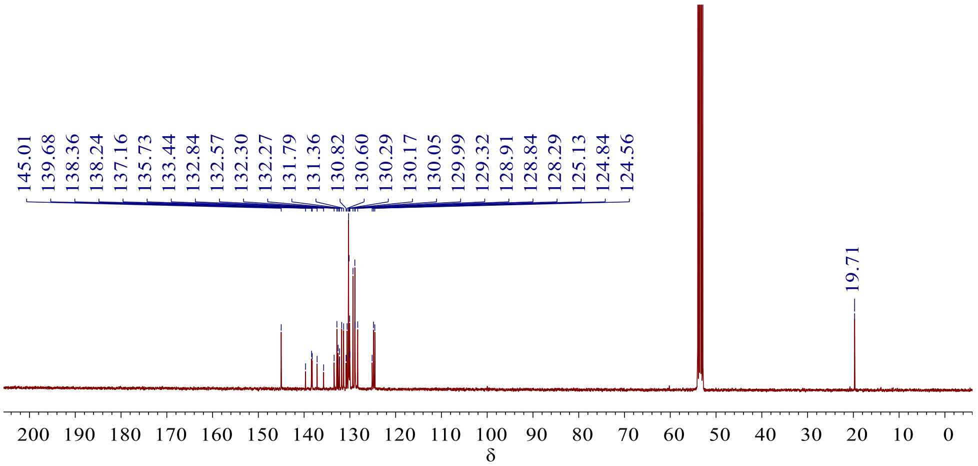

Figure S6. ^{13}C NMR spectrum of compound *c*$_5$-TPBQ in CD_2Cl_2.

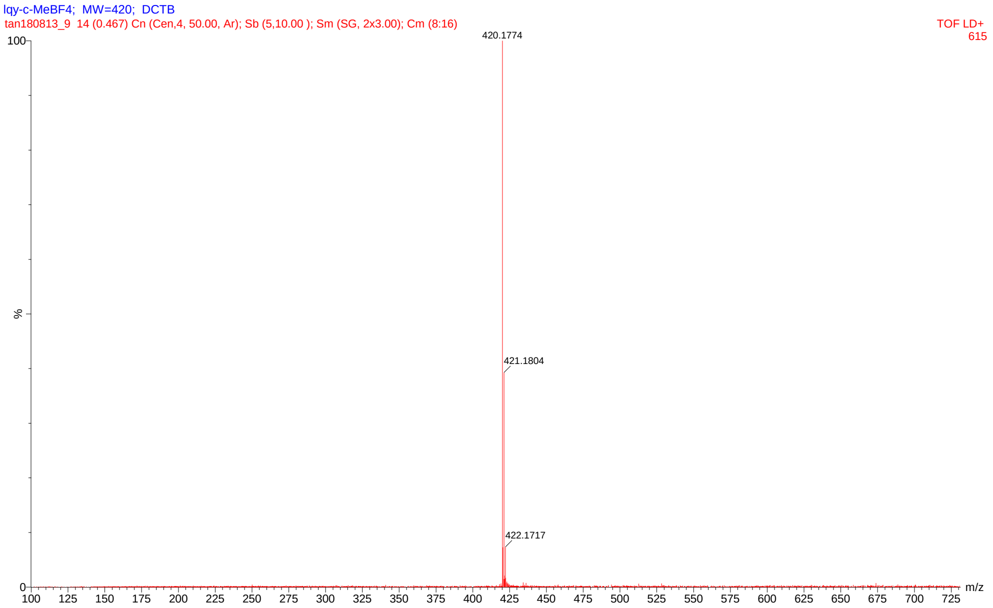

Figure S7. HRMS spectrum of compound *c*$_5$-TPBQ.

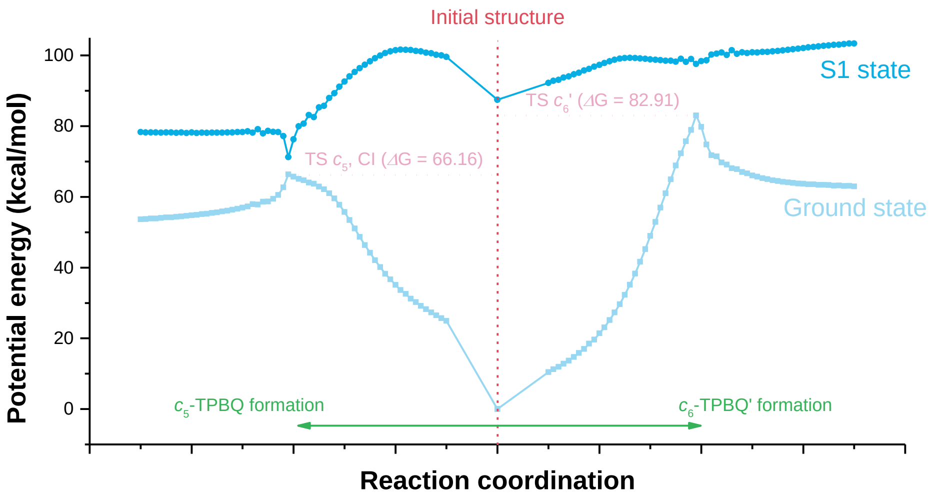

Figure S8. Potential energy surface of ground and S1 state on six-membered and five-membered ring products formation process.

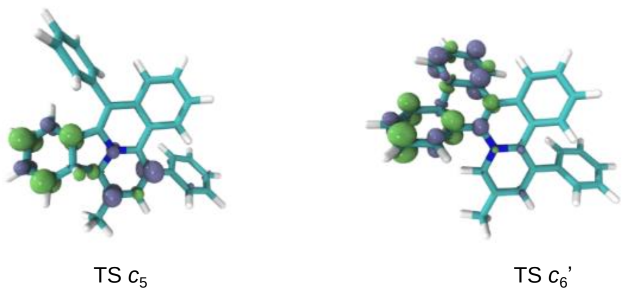

Figure S9. Iso-surface (iso-value=0.01) on spin density of TS c_5 and TS c_6'.

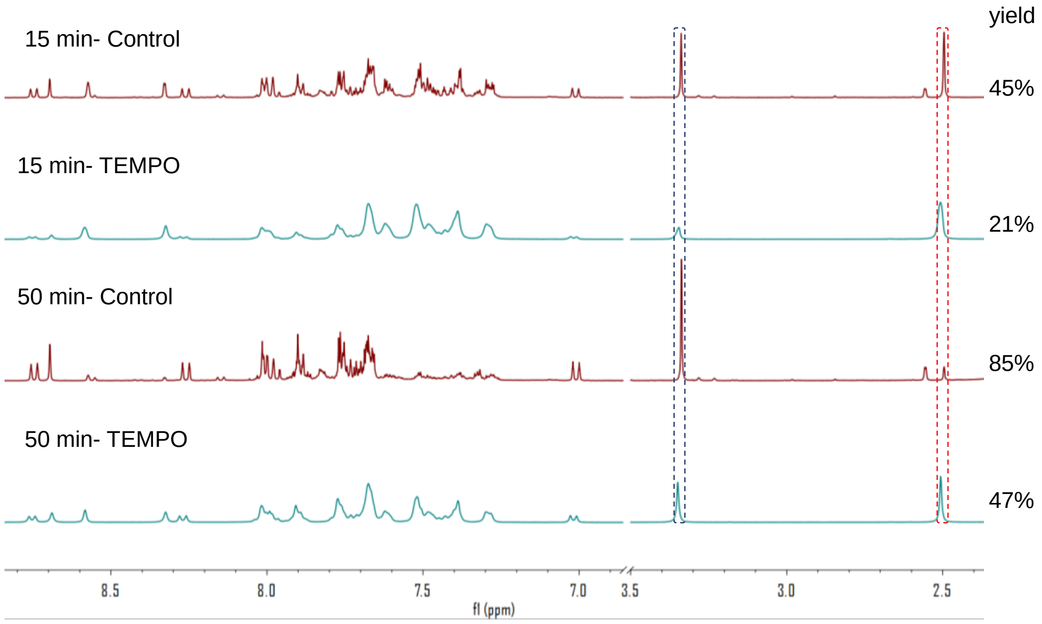

Figure S10. Time-dependent ^{1}H NMR spectra of *o*-TPBQ in CD_3CN solution under UV irradiation. TEMPO used: 3 equiv.

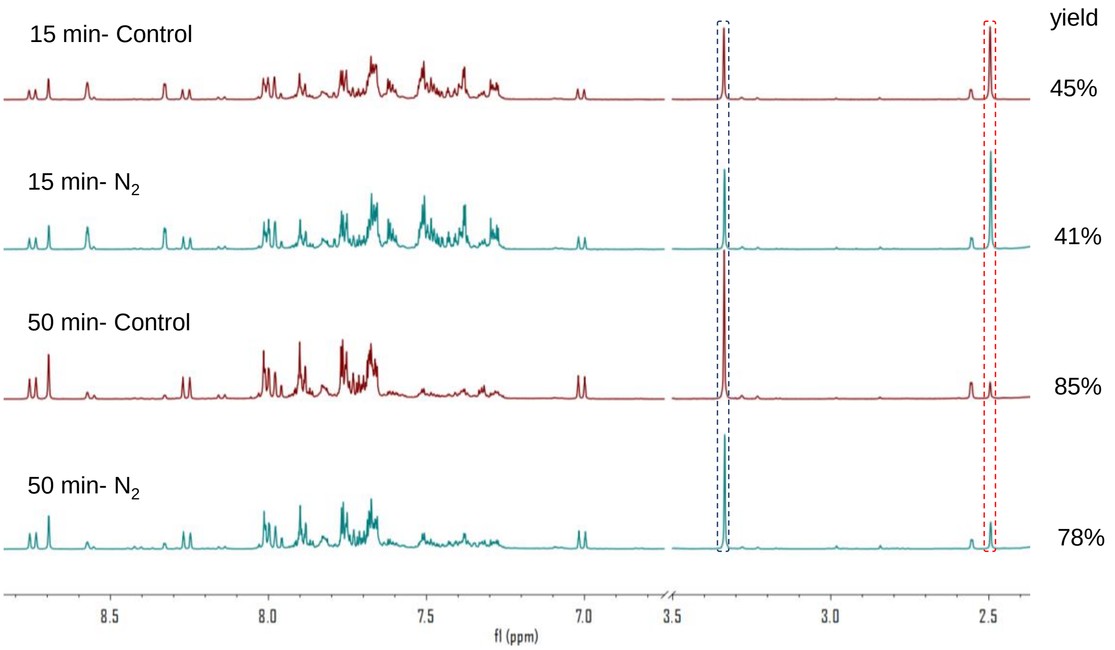

Figure S11. Time-dependent ^{1}H NMR spectra of *o*-TPBQ in CD_3CN solution under UV irradiation.

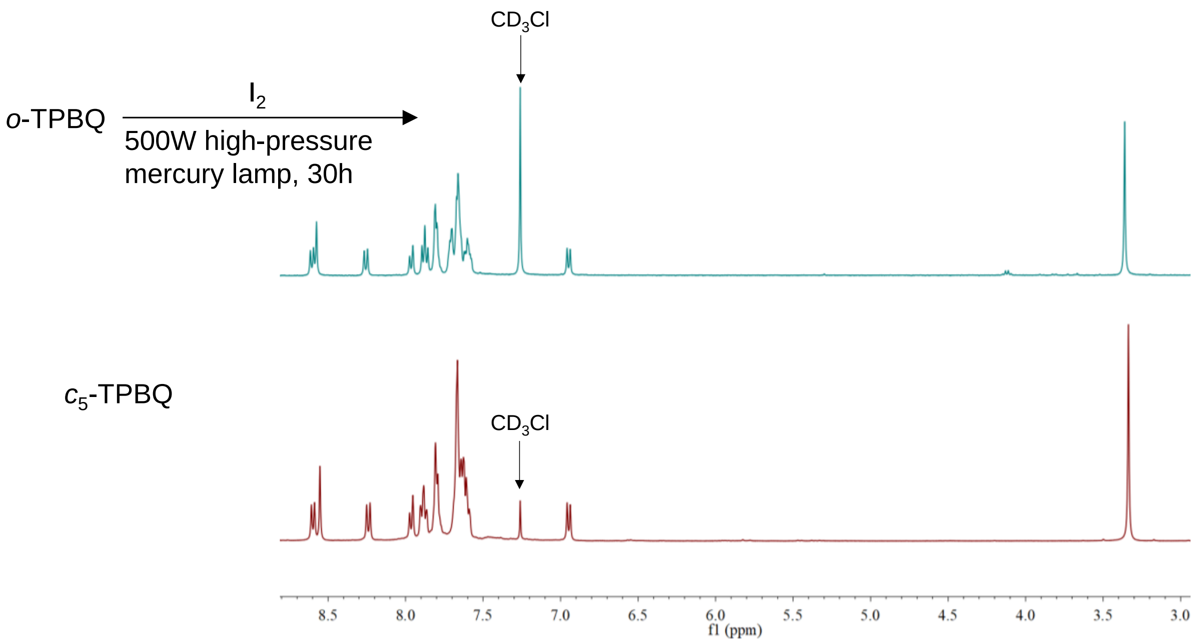

Figure S12. ^{1}H NMR spectra of *o*-TPBQ in CD_3Cl solution after irradiation from a 500 W high-pressure mercury lamp for 30 h and ^{1}H NMR spectra of c_5-TPBQ.

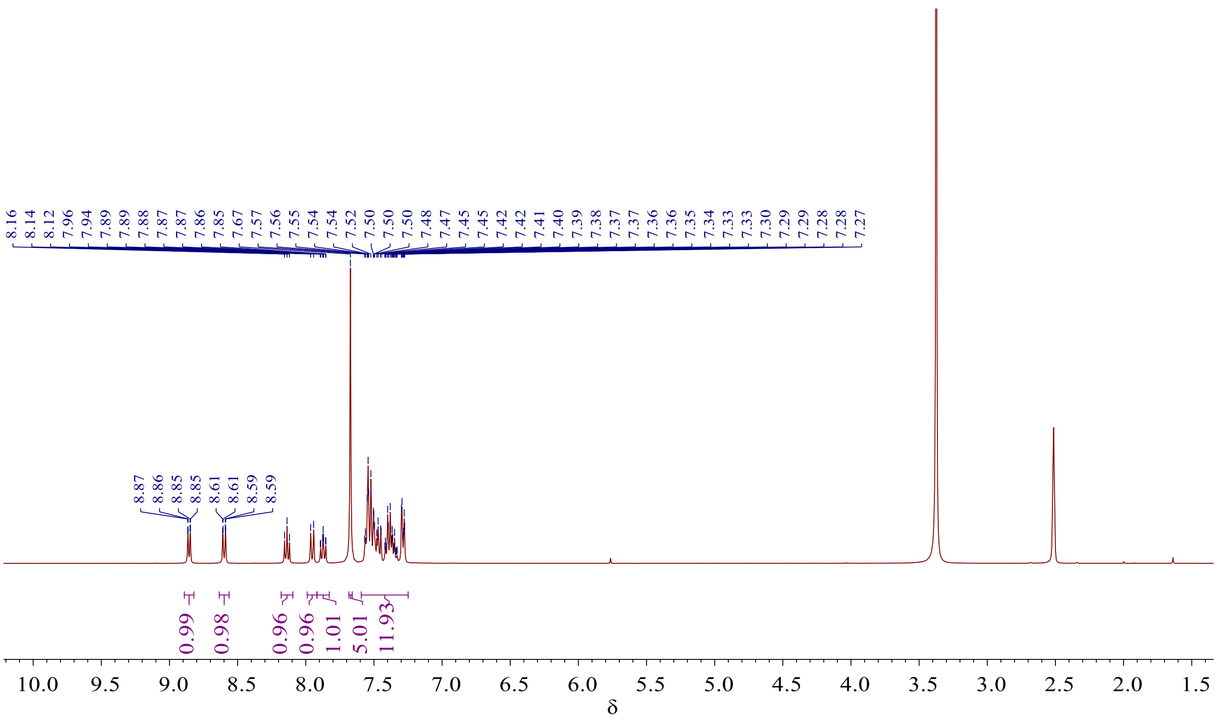

Figure S13. ^{1}H NMR spectrum of compound *o*-I in DMSO-d_6

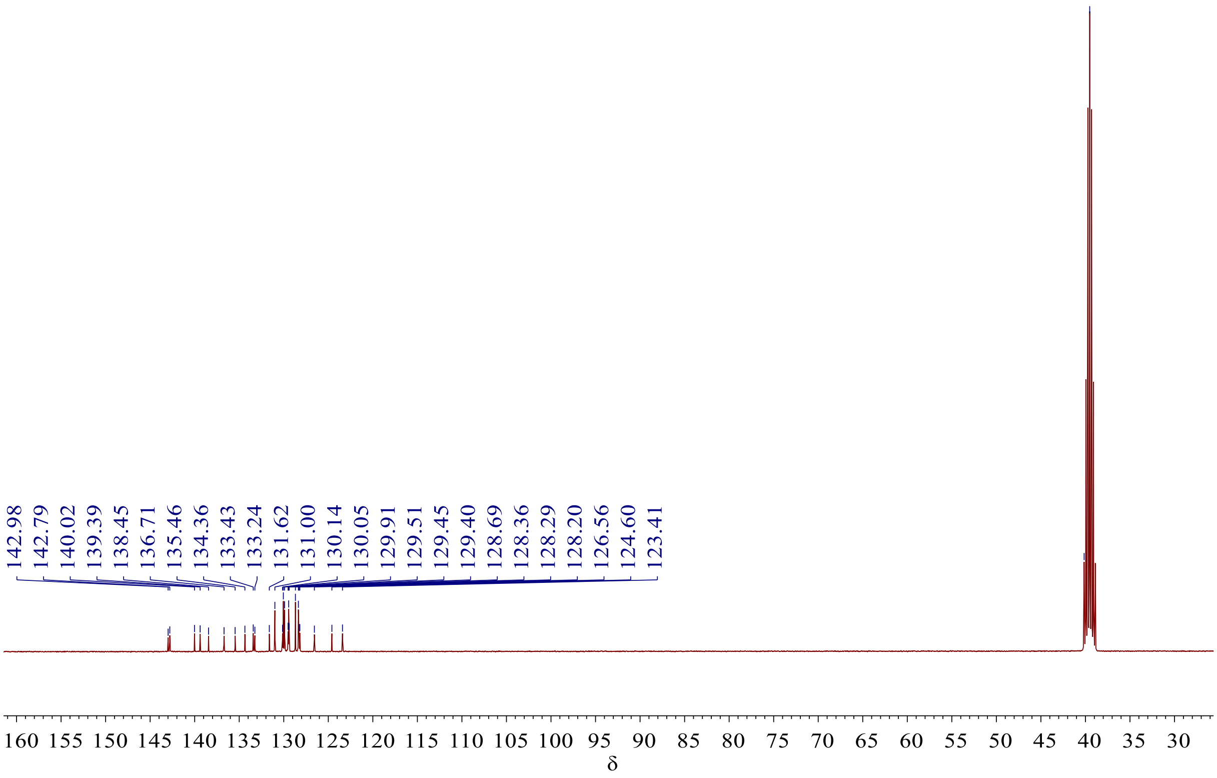

Figure S14. ^{13}C NMR spectrum of compound *o*-I in DMSO-d_6.

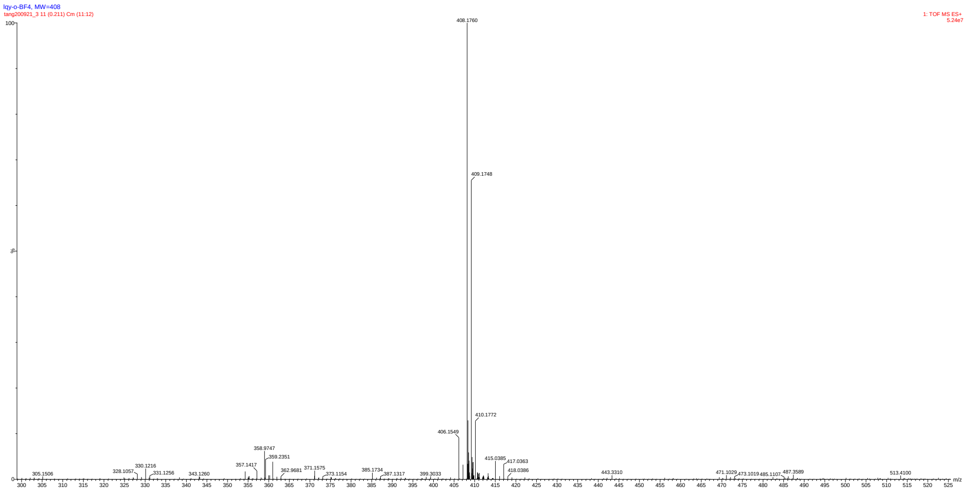

Figure S15. HRMS spectrum of compound *o*-I.

Table S3. Crystallographic and structural refinement data of *o*-I.[a]

Empirical formula	$C_{31}H_{22}BF_4N$
Formula weight	495.30
Temperature (K)	100.15
Wavelength (Å)	1.54184
Crystal system	Triclinic
space group	P-1
a (Å)	8.4537(6)
b (Å)	11.0248(9)
c (Å)	13.8145(10)
α (°)	108.087(7)
β (°)	95.552(6)
γ (°)	99.640(6)
Volume (Å^3)	1191.31(16)
Z	2
θ range (°)	3.409 to 67.494
Index ranges	-10<=h<=10,-8<=k<=13,-6<=l<=16

[a]Crystallographic data for the structures reported in this paper have been deposited with the Cambridge Crystallographic Data Centre as supplementary publication no. CCDC: 2035826 for *o*-I.

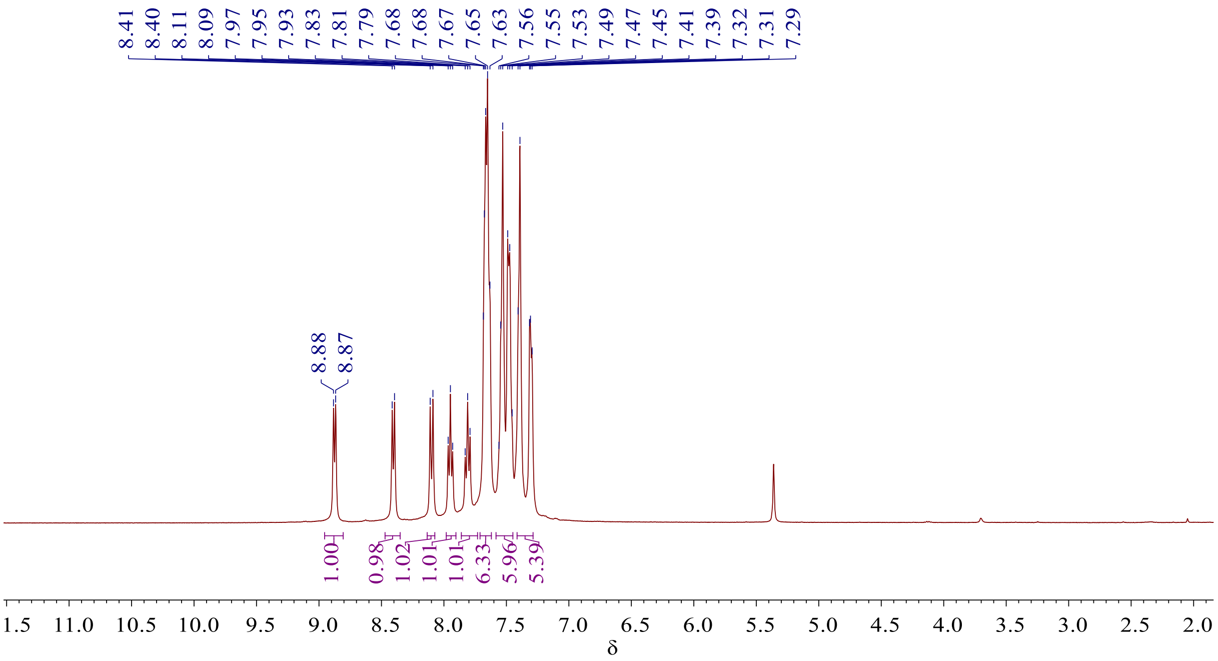

Figure S16. ^{1}H NMR spectrum of compound *o*-II in CD_2Cl_2

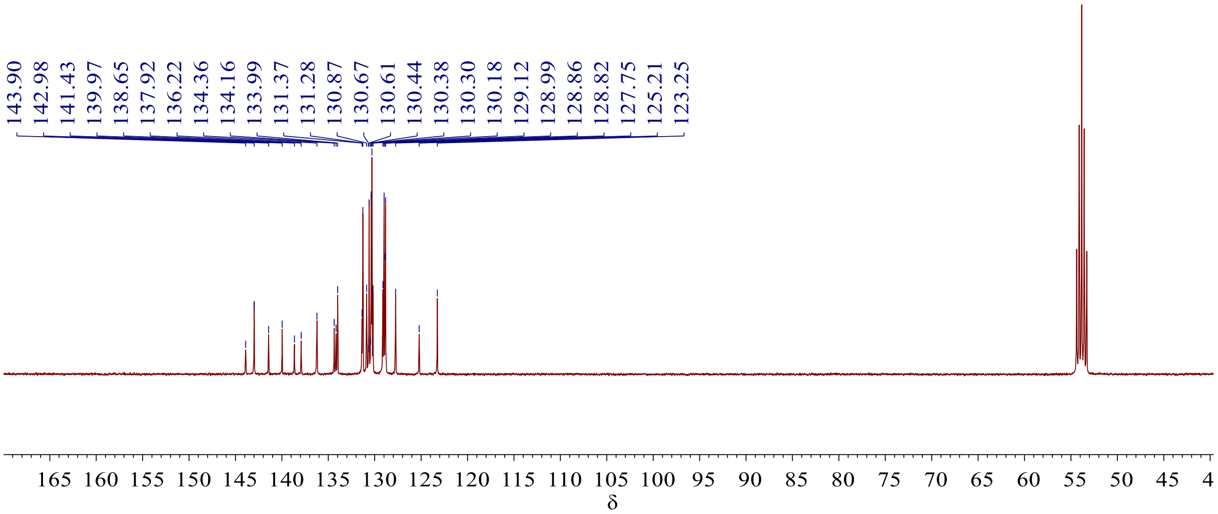

Figure S17. ^{13}C NMR spectrum of compound *o*-II in CD_2Cl_2

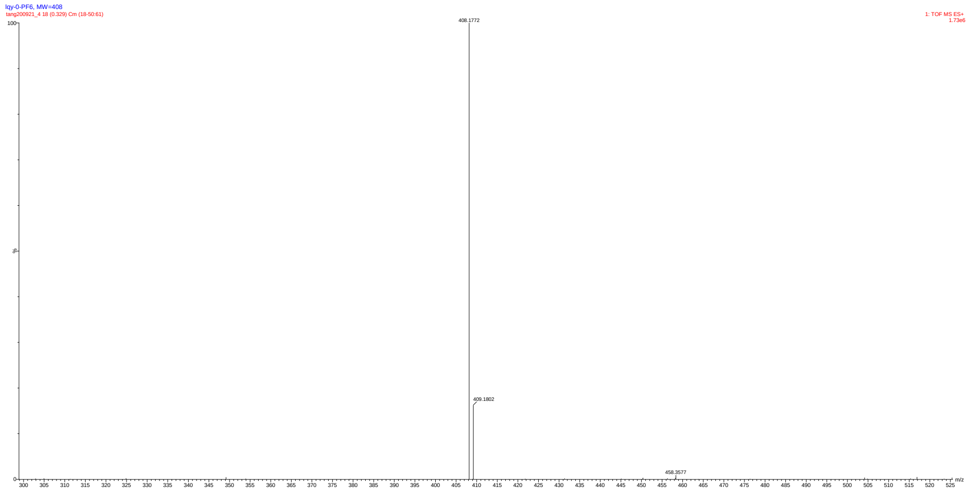

Figure S18. HRMS spectrum of compound *o*-II.

Table S4. Crystallographic and structural refinement data of *o*-II.[a]

Empirical formula	$C_{31}H_{22}F_6NP$
Formula weight	553.46
Temperature (K)	100.01(10)
Wavelength (Å)	1.54184
Crystal system	triclinic
space group	P-1
a (Å)	8.5484(5)
b (Å)	11.4387(6)
c (Å)	13.6080(9)
α (°)	103.553(5)
β (°)	90.551(5)
γ (°)	98.266(5)
Volume (Å^3)	1278.83(14)
Z	2
θ range (°)	4.021 to 67.488
Index ranges	$-10 \le h \le 7$, $-13 \le k \le 13$, $-16 \le l \le 15$

[a]Crystallographic data for the structures reported in this paper have been deposited with the Cambridge Crystallographic Data Centre as supplementary publication no. CCDC: 2035839 for *o*-II.

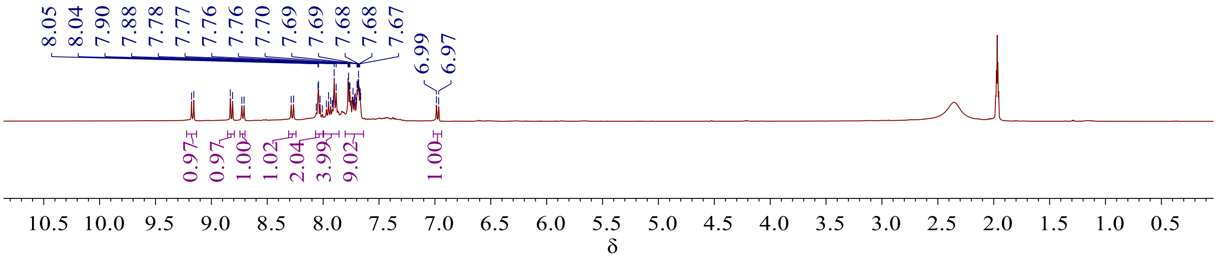

Figure S19.^{1}H NMR spectrum of compound c_5-I in CD_3CN.

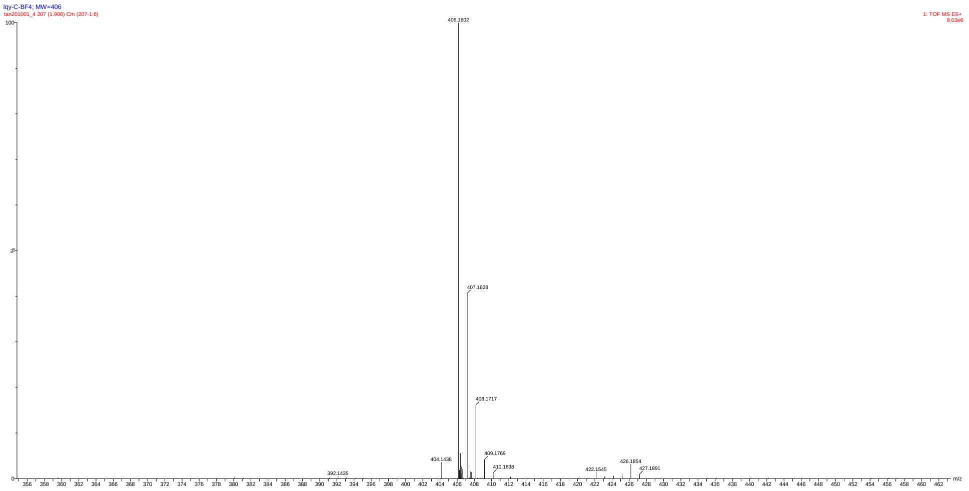

Figure S20. HRMS spectrum of compound c_5-I.

Table S5. Crystallographic and structural refinement data of *c*5-I.[a]

Empirical formula	$C_{33}H_{22}BCl_6F_4N$
Formula weight	732.02
Temperature (K)	100.15
Wavelength (Å)	1.54184
Crystal system	Monoclinic
space group	P 1 21/c 1
a (Å)	11.07767(20)
b (Å)	21.6410(4)
c (Å)	13.7711(3)
α (°)	90
β (°)	101.8388(18)
γ (°)	90
Volume (Å^3)	3231.14(10)
Z	4
θ range (°)	3.864 to 67.500
Index ranges	-13<=h<=13,-18<=k<=25,-16<=l<=16

[a]Crystallographic data for the structures reported in this paper have been deposited with the Cambridge Crystallographic Data Centre as supplementary publication no. CCDC: 2035827 for *c*5-I.

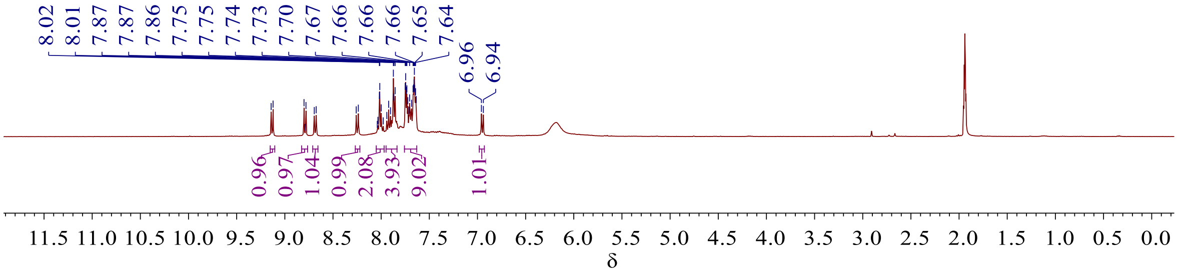

Figure S21.^{1}H NMR spectrum of compound *c*5-II in CD_3CN.

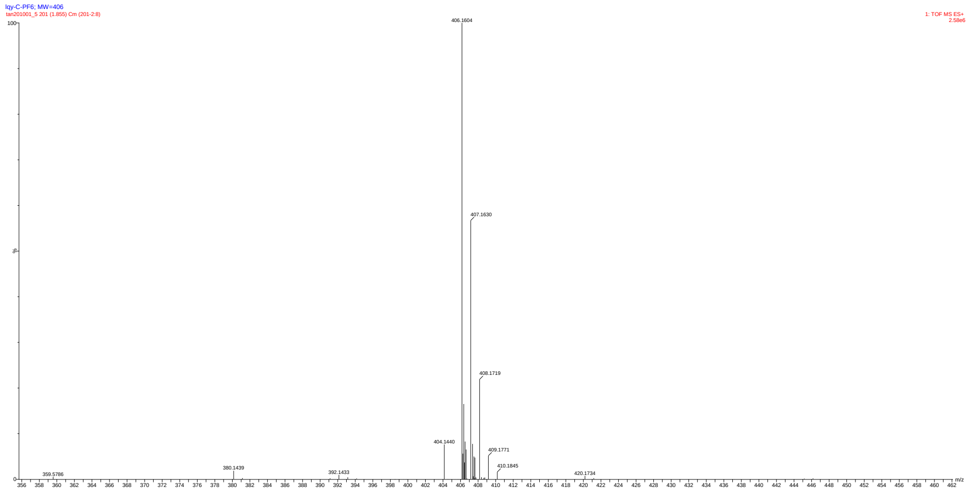

Figure S22. HRMS spectrum of compound c_5-II.

Table S6. Photophysical properties of *o*-TPBQ and c_5-TPBQ.

compound	λ_{abs} (nm)	solution	aggregate			α_{AIE}[b]	solid		
		Φ (%)	λ_{ex} (nm)	Φ (%)	τ (ns)[a]		λ_{ex} (nm)	Φ (%)	τ (ns)[a]
o-TPBQ	380	1.8	450	21.6	4.48	12.0	435, 470	12.5	5.29
c_5-TPBQ	390	1.1	460	63.3	11.12	57.5	490	12.3	3.43

[a]τ is defined as average fluorescence lifetime calculated by $\tau = \Sigma A_i(\tau_i)^2/\Sigma A_i\tau_i$, where A_i is the pre-exponential factor for lifetime. [b]α_{AIE} is defined as $\Phi_{aggregate}/\Phi_{solution}$.

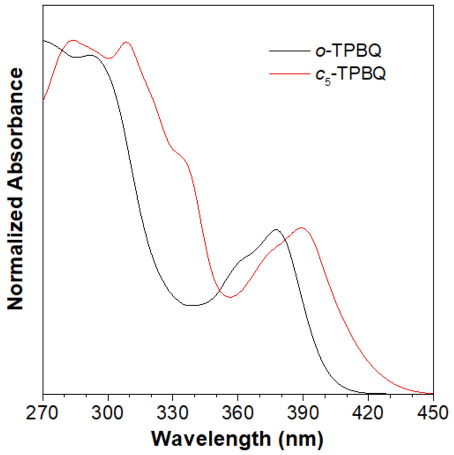

Figure S23. UV-vis spectra of *o*-TPBQ and c_5-TPBQ in DMSO solution. Concentration: 10 μM.

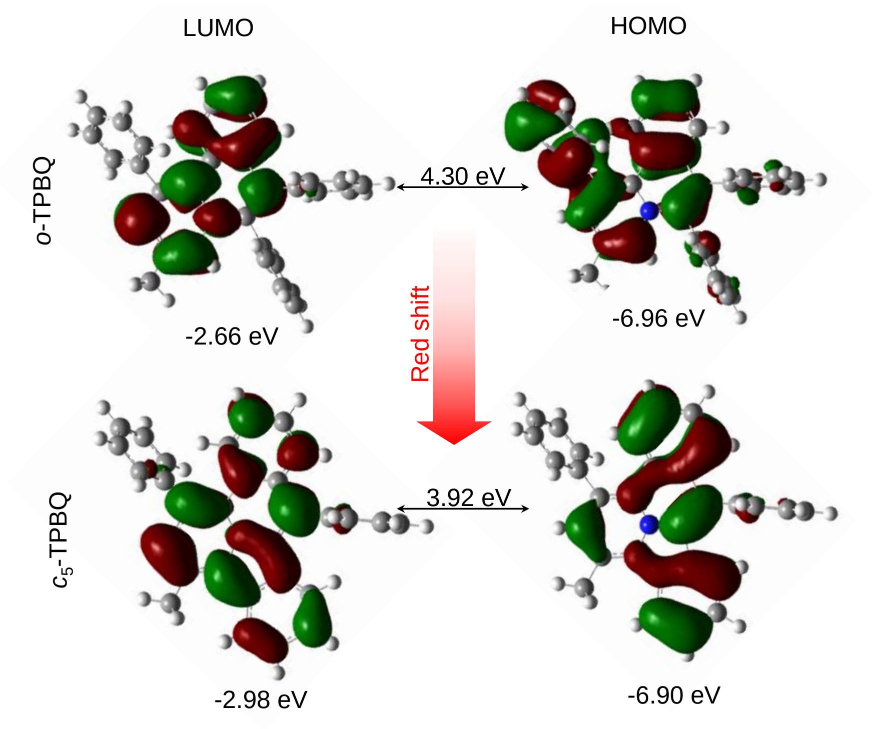

Figure S24. LUMO and HOMO orbital distributions of *o*-TPBQ and c_5-TPBQ.

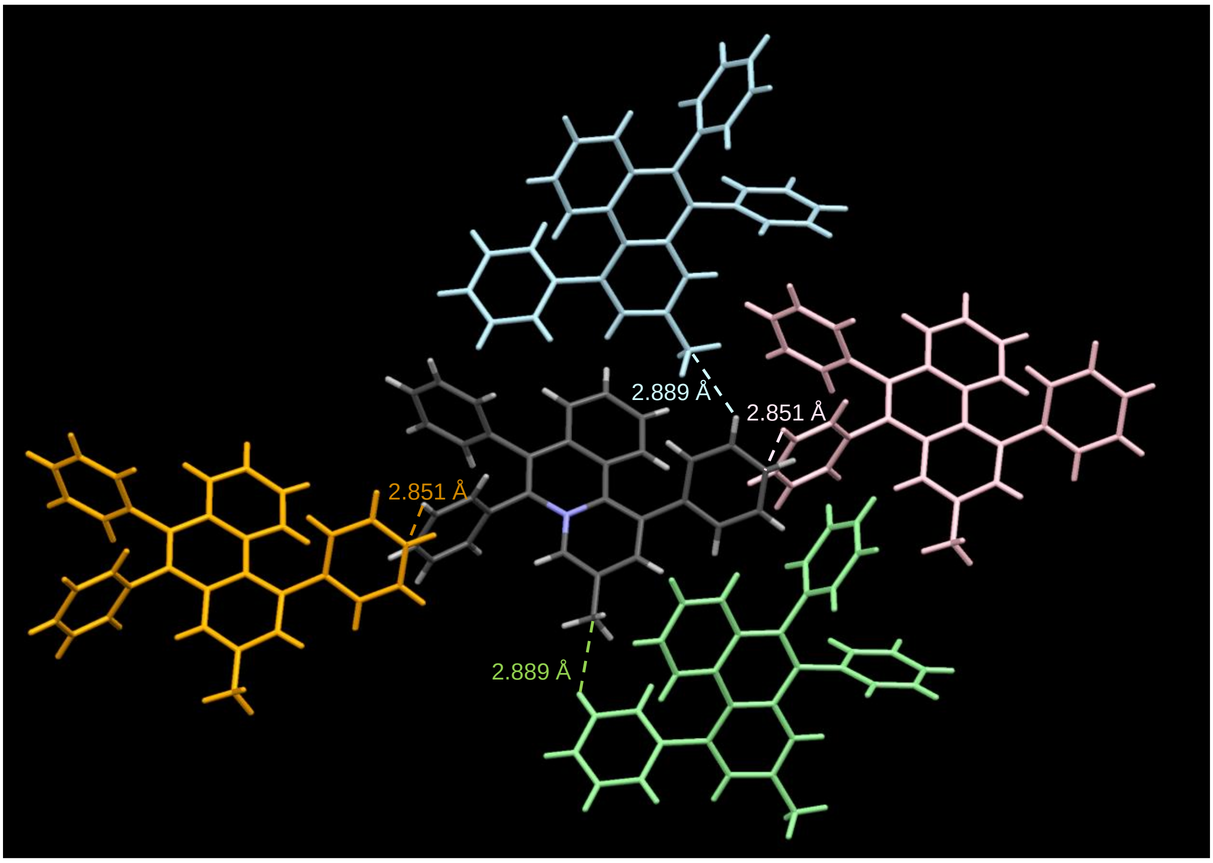

Figure S25. Short contact interactions in the crystal structure of *o*-TPBQ.

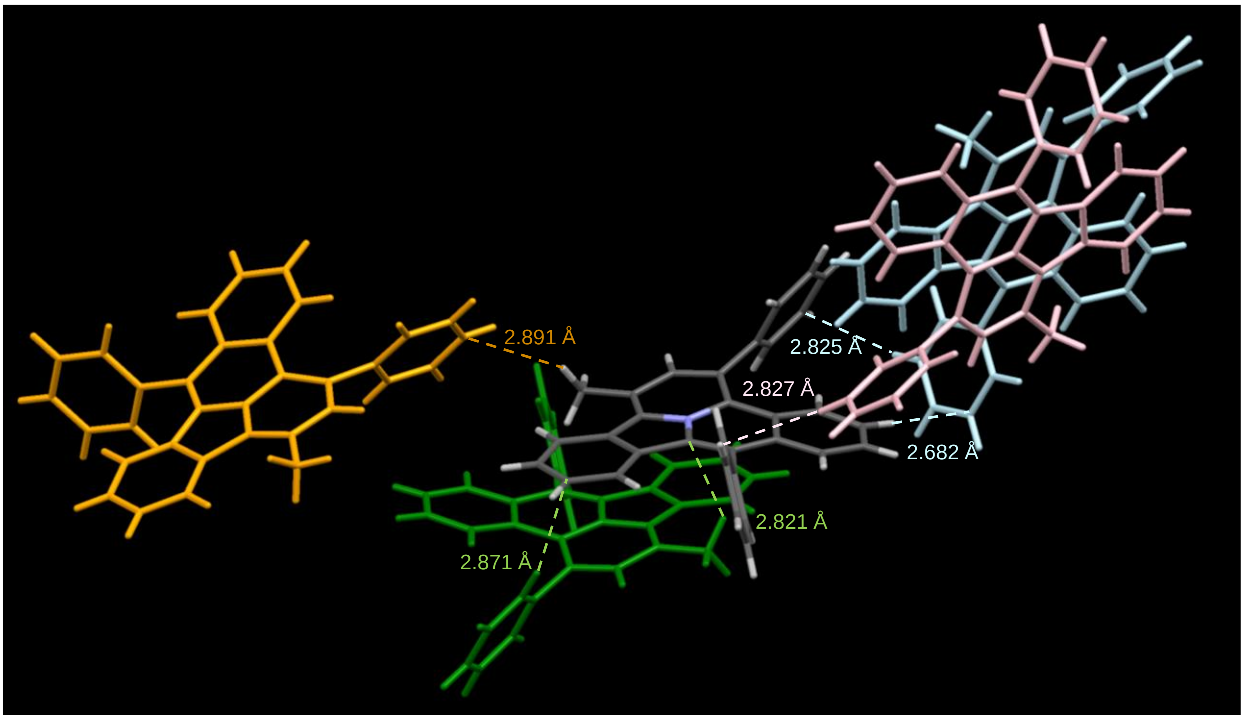

Figure S26. Short contact interactions in the crystal structure of c_5-TPBQ.

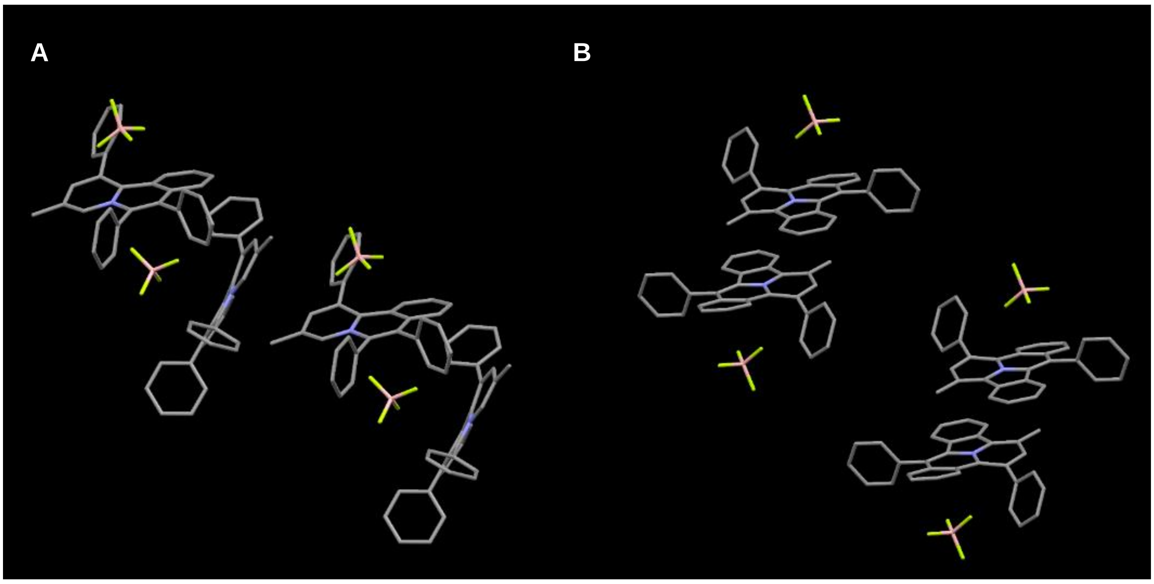

Figure S27. Crystal packing of *o*-TPBQ (A) and c_5-TPBQ (B).

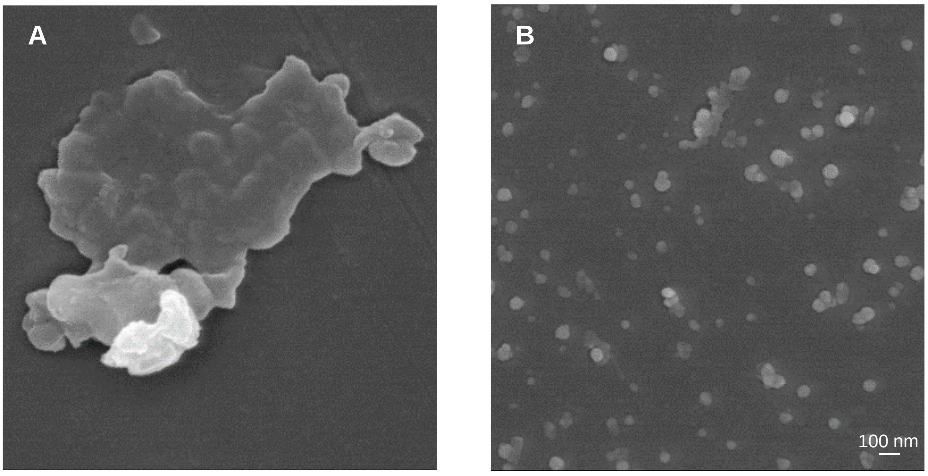

Figure S28. SEM images of *o*-TPBQ (A) and c_5-TPBQ (B) in aggregate state, respectively.

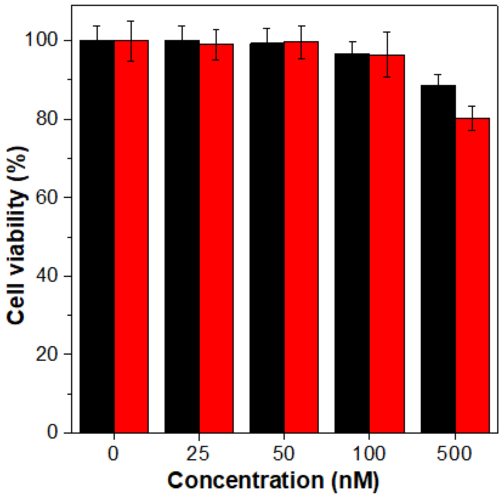

Figure S29. Cell viability of Hela cells incubated with *o*-TPBQ (black) and c_5-TPBQ (red).

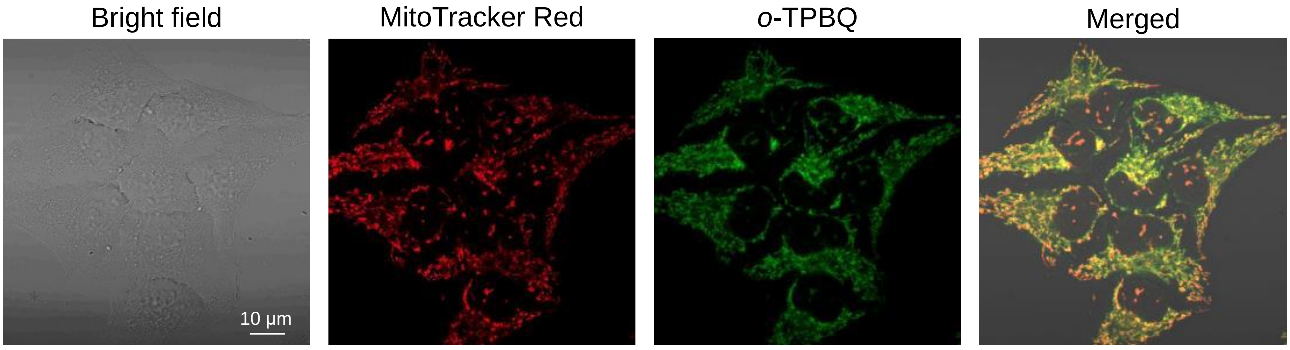

Figure S30. Co-localization imaging of Hela cancer cells. Concentration: 500 nM.

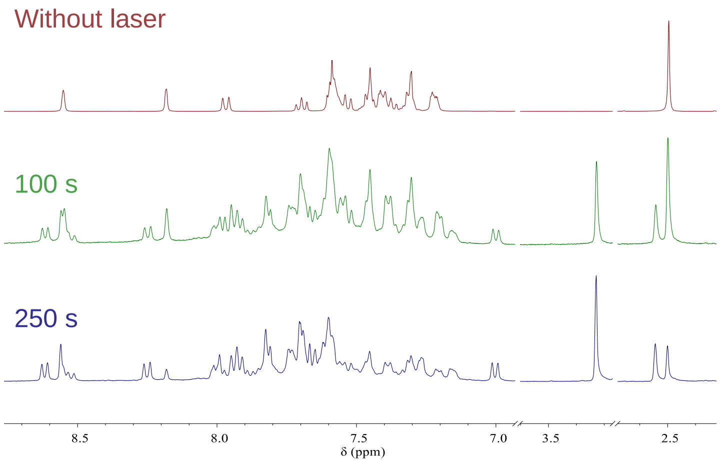

Figure S31. ^{1}H NMR spectra of *o*-TPBQ dispersed in cell medium under laser irradiation.

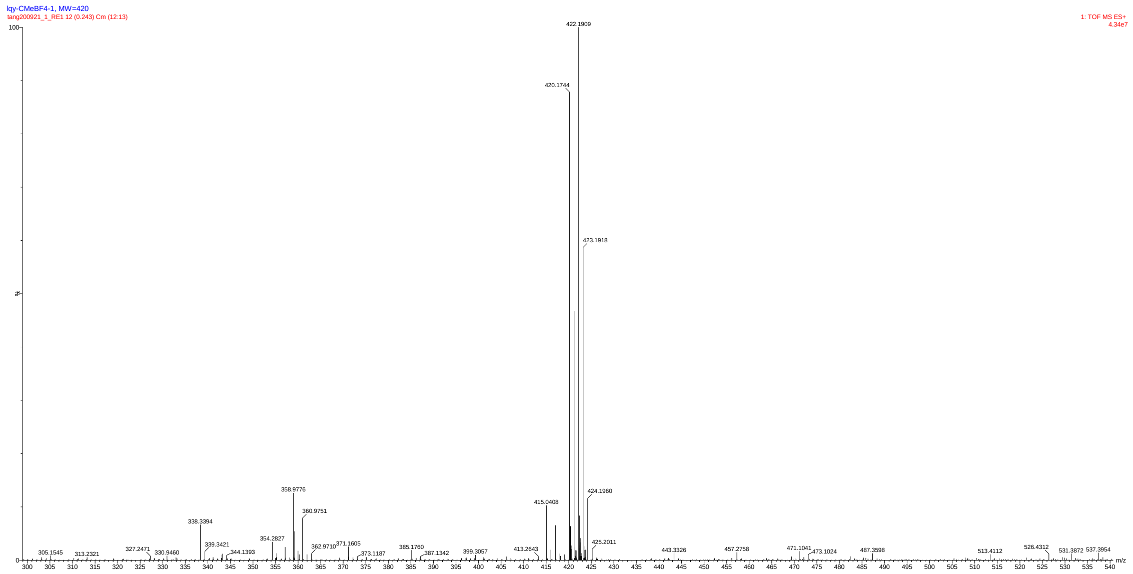

Figure S32. HRMS spectrum of *o*-TPBQ dispersed in cell medium after laser irradiation.

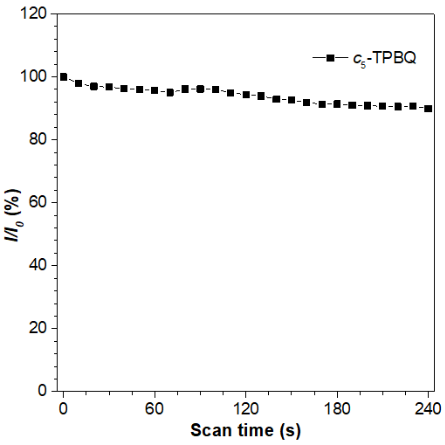

Figure S33. Loss in fluorescence of HeLa cells stained with c_5-TPBQ with increasing scan time.

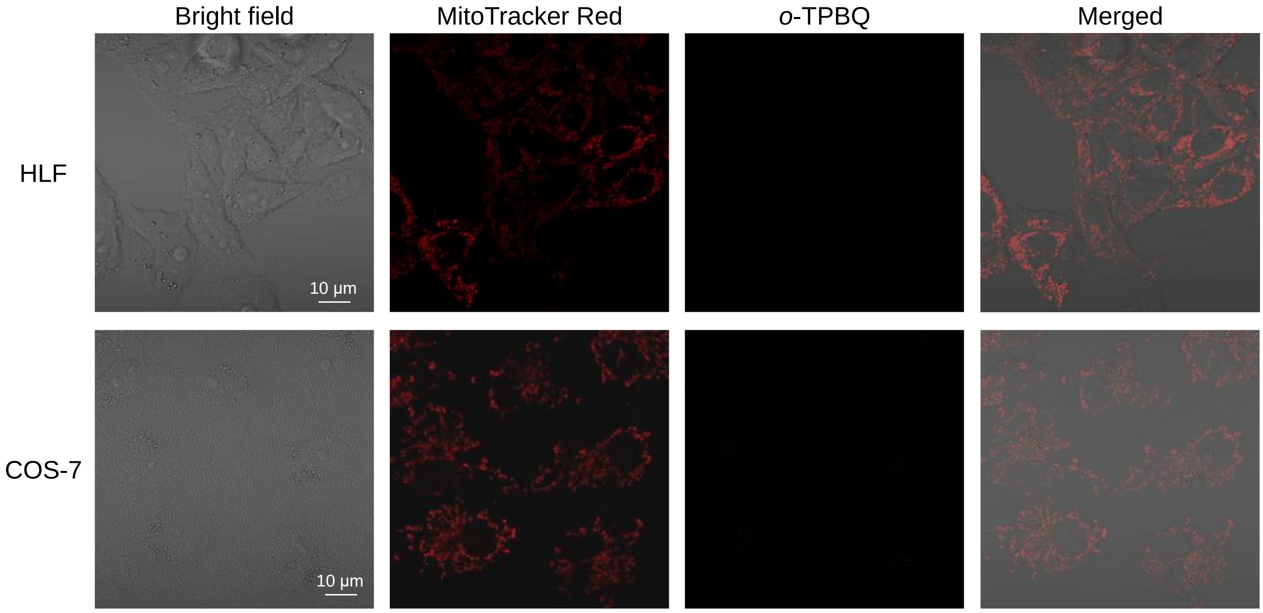

Figure S34. Co-localization imaging of normal cells. Concentration: 50 nM.

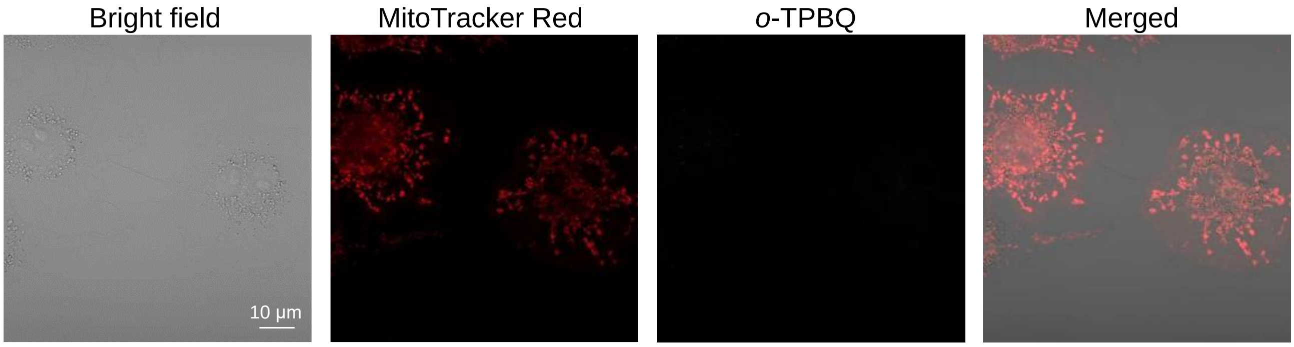

Figure S35. Co-localization imaging of COS-7 normal cells. Concentration: 1 μM.

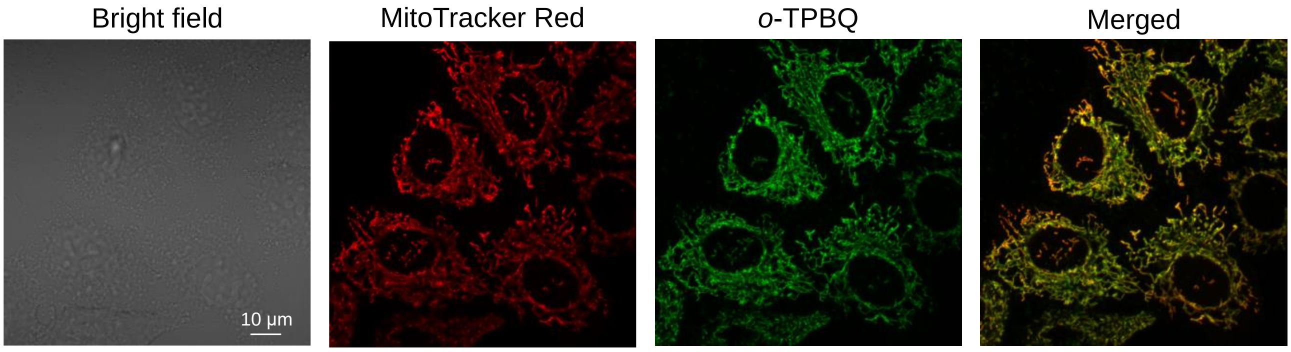

Figure S36. Co-localization imaging of HepG2 cancer cells. Concentration: 500 nM.

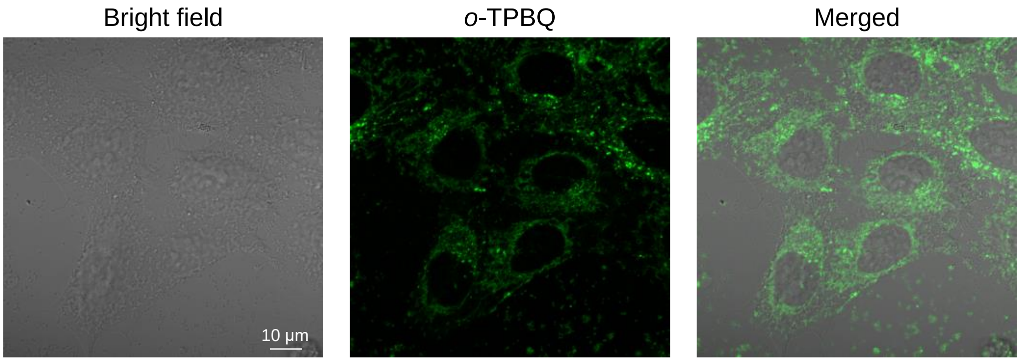

Figure S37. Imaging of HepG2 cancer cells. Concentration: 50 nM.

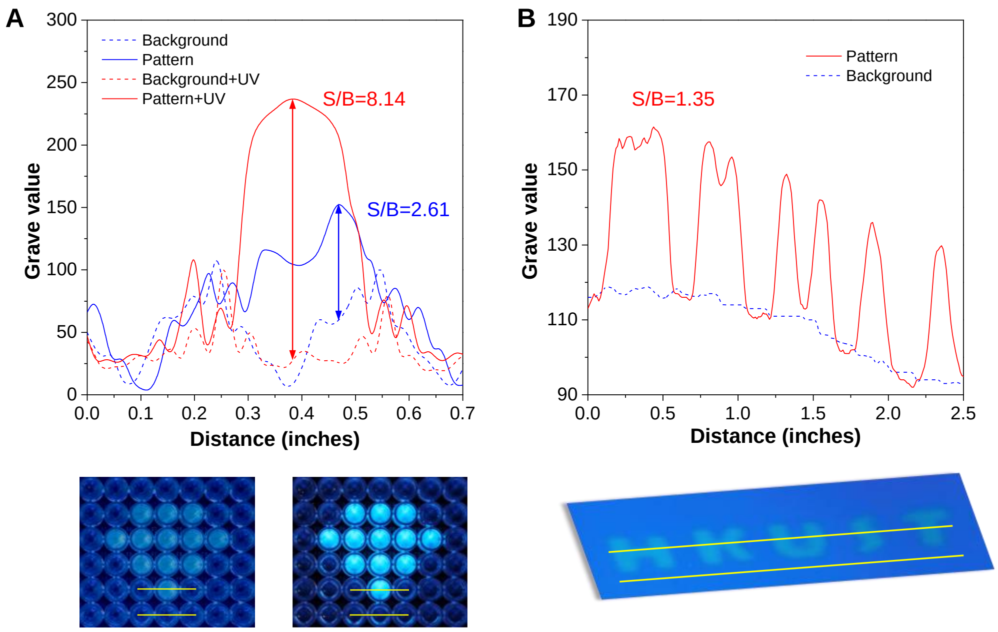

Figure S38. The contrast intensity of pattern and background area corresponding to the yellow line before and after UV irradiation.
